# Highly Pathogenic Avian Influenza A(H7N3) Virus in Poultry, United States, 2020

**DOI:** 10.3201/eid2612.202790

**Published:** 2020-12

**Authors:** Sungsu Youk, Dong-Hun Lee, Mary L. Killian, Mary J. Pantin-Jackwood, David E. Swayne, Mia K. Torchetti

**Affiliations:** Southeast Poultry Research Laboratory, US Department of Agriculture Agricultural Research Service, Athens, Georgia, USA (S. Youk, M. Pantin-Jackwood, D. Swayne);; University of Connecticut, Storrs, Connecticut, USA (D.-H. Lee); US Department of Agriculture, Ames, Iowa, USA (M. Killian, M. Torchetti)

**Keywords:** highly pathogenic avian influenza virus, influenza A(H7N3), poultry, phylogenetic analysis, United States, influenza, viruses

## Abstract

An outbreak of low-pathogenicity avian influenza A(H7N3) virus of North American wild bird lineage occurred on commercial turkey farms in North Carolina and South Carolina, USA, during March–April 2020. The virus mutated to the highly pathogenic form in 1 house on 1 farm via recombination with host 28S rRNA.

Highly pathogenic avian influenza viruses (HPAIVs) have devastating impacts on the poultry industries. With infections in poultry, H5 and H7 low-pathogenicity avian influenza viruses (LPAIVs) have spontaneously mutated into HPAIVs by different mechanisms, one of which is acquisition of basic amino acids at the hemagglutinin (HA) cleavage site (*1*).

In March 2020, an outbreak of LPAIV H7N3 occurred in turkey farms, affecting 11 premises in North Carolina and 1 in South Carolina, USA. The initial decision to depopulate LPAIV-affected flocks was based on a risk assessment that included the location of affected premises, the poultry density in the area, and the presence of a basic amino acid substitution at the cleavage site among the initial LPAIV detections (PEKPKTR/GLF; substitution sequence is underscored). During the ongoing response for this event, the Clemson Veterinary Diagnostic Center in Columbia, South Carolina, a member of the National Animal Health Laboratory Network, detected an influenza A(H7) outbreak in a second turkey location in South Carolina, with increased death and respiratory signs; oropharyngeal and cloacal swab samples were forwarded to the National Veterinary Services Laboratories in Ames, Iowa, USA. On April 8, the National Veterinary Services Laboratories confirmed 1 of 6 pooled samples as HPAIV H7N3. Subsequent testing from all infected barns on the premises determined that HPAIV was present in only 1 of the 5 barns, but LPAIV was identified in the other 4 barns. All the premises affected by LPAIV and HPAIV H7N3 were located in 3 adjacent counties and 1 across state lines, indicating that geographic proximity was relevant to the outbreaks. Immediate depopulation was performed on the LPAIV- and HPAIV-affected premises, affecting 361,000 birds.

Complete genome sequencing and phylogenetic analyses were conducted to trace the origin and evolution of the H7N3 viruses. A total of 29 H7N3 viruses from 13 premises were sequenced (Appendix). Complete genome sequences have been deposited in GenBank (accession nos. MT444183–350 and MT444352–415). The intravenous pathogenicity index of selected LPAIV strains was 0 and of selected HPAIV strains, 2.46. Based on the HA cleavage site motif and supported by the intravenous pathogenicity index, 2 H7N3 viruses from 1 house in South Carolina were considered to be HPAIV. For the 2 HPAIVs, 34 (5.7%) and 1,076 (38.8%) reads had no insertion, whereas the rest of the reads were found to have an identical 27 nucleotides insertion from host cellular 28S rRNA in the cleavage site. 

The presence of LPAIV and HPAIV in 1 barn at the same time suggests that the mutation was caught early. The 27-nt insertion coding for 9 amino acids at the HA cleavage site (PENPKTDRKSRHRRIR/GLF; insertion sequence is underscored) is identical to that found in a 2017 HPAIV H7N9 from a poultry outbreak that occurred in Tennessee (*2*). The potential role of a palindromic sequence was suggested to be a cause of RNA recombination with host 28S rRNA (*3*), and similar structure is often observed among the H7 subtype (*4*); however, the exact mechanism of recombination for this particular insert remains to be elucidated. Another notable change was a 66-nt deletion in the NA stalk region from 2 premises in North Carolina. The NA stalk deletions are commonly associated with adaptation of wild bird avian influenza viruses to gallinaceous poultry (*5*); for these isolates, all reads had the same 66-nt deletion (e.g., no mixed population was detected). No significant amino acid change indicating adaptation to mammalian hosts was identified by using the Influenza Research Database (Appendix).

Maximum-likelihood and Bayesian relaxed clock phylogenies of each gene were generated (*6*,*7*). All phylogenies indicate that the North Carolina and South Carolina H7N3 viruses were genetically distinct from recent Mexico H7N3 HPAIV, 2016 Indiana H7N8, and 2017 Tennessee H7N9 poultry outbreak strains (Appendix Figures 1, 2) (*2*,*8*,*9*). All H7N3 viruses from North Carolina and South Carolina clustered together in phylogenies across all 8 gene segments and showed high levels of nucleotide identity (>99.28%). These data support a single source of LPAIV H7N3 being introduced to turkey farms in North Carolina, spreading laterally to other turkey premises, and mutating once to HPAIV during replication in turkeys from a single barn on a turkey premises in South Carolina, with no further reassortment with any other influenza strains. Furthermore, the H7 HA genes of recent US poultry events (2016, 2017, and 2020) originate from the same North American wild bird H7 clade. With several recent incursions, this H7 HA clade represents a repetitive threat to domestic poultry and carries with it the potential to mutate to HPAIV.

**Figure 1:**
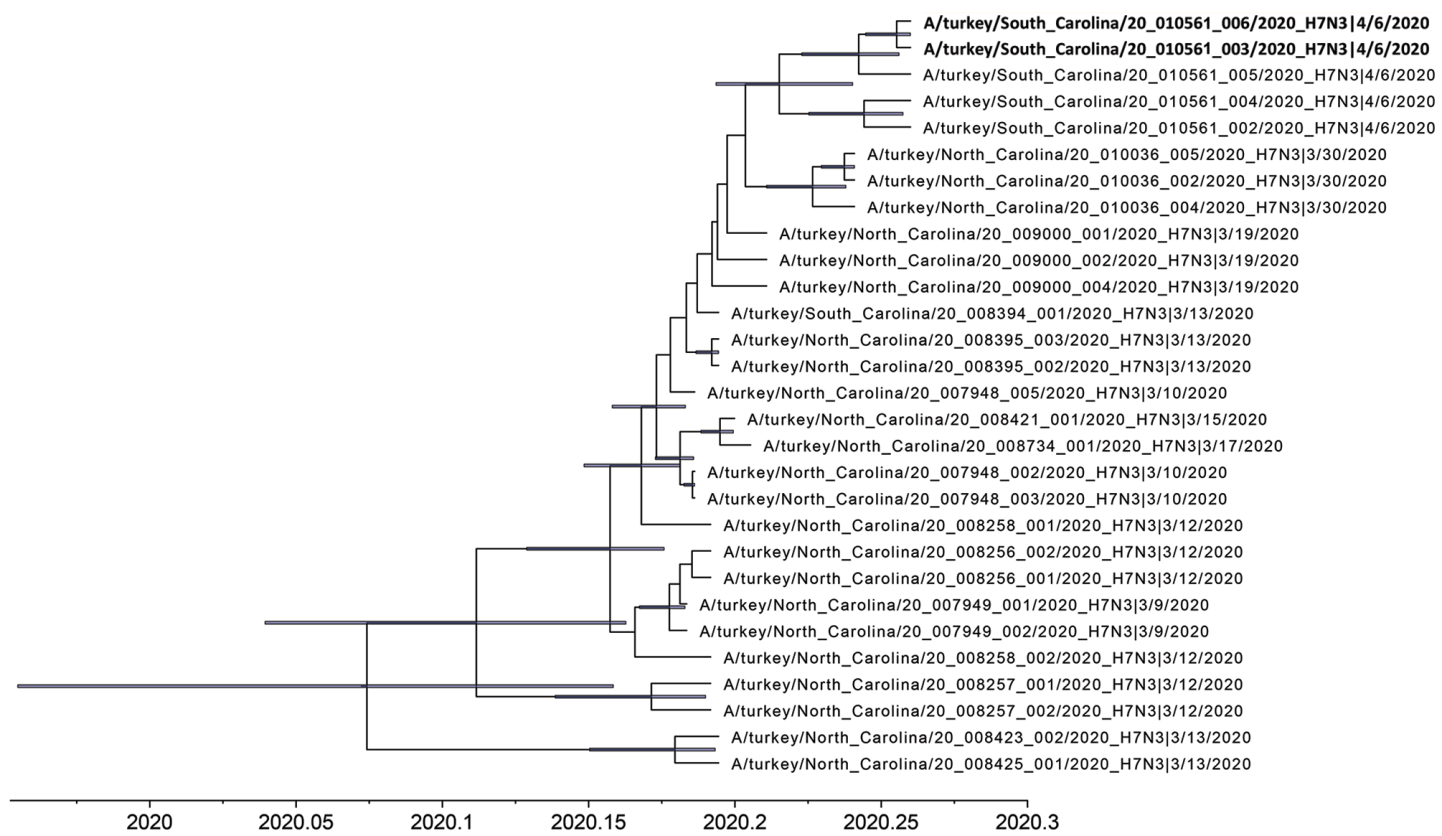
Time-scaled Bayesian maximum clade credibility tree of the concatenated whole genome of highly pathogenic avian influenza A(H7N3) viruses from South Carolina (bold) and North Carolina, USA. Node bars represent 95% Bayesian credible intervals for estimates of common ancestry.

**Figure 2:**
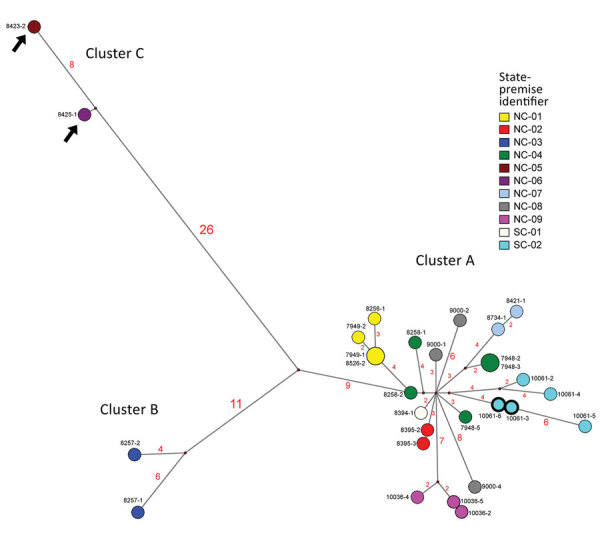
Median-joining phylogenetic network of the concatenated whole genome of highly pathogenic avian influenza (HPAIV) A(H7N3) viruses from South Carolina and North Carolina , USA. This network tree includes all the most parsimonious trees linking the sequences. Each unique sequence is represented by a circle sized relative to its frequency. The number of nucleotide differences between viruses is indicated on the branches. Isolates are colored according to the source premises. The black arrows indicate the H7N3 viruses with the 66-nt deletion at the neuraminidase stalk region (the NA of these 2 viruses was modified to exclude the deletion for this analysis). Both low-pathogenicity (turquoise) and HPAIV (turquoise with bolded black outline) viruses were recovered from the SC-02 premises (the hemagglutinin of the 2 HPAIVs excludes the insertion for this analysis).

To increase phylogenetic resolution, nucleotide sequences from the entire protein coding regions of each virus were concatenated, with 2 modifications: the insertion at the HA cleavage site for the HPAIV was excluded, and the deletion at the NA stalk region for the 2 LPAIVs was removed. The concatenated sequences were then analyzed with Bayesian and median-joining network phylogenetic analysis (*7*,*10*). This analysis highlights that the LPAIV H7N3 diverged early into 3 well-supported genetic clusters (A, B, and C) with high posterior probabilities (posterior probability [PP] >0.99) (Figure 1). Cluster A was detected most frequently from 10 premises in both North Carolina and South Carolina and mutated to HPAIV in a single turkey location in South Carolina (Figure 2). The estimated mean time to most recent common ancestor (tMRCA) of the concatenated whole genome of H7N3 viruses from North Carolina and South Carolina was January 25, 2020 (95% Bayesian credible interval [BCI] December 14, 2019–February 28, 2020; PP = 1.0). The tMRCA, in addition to the NA stalk deletion indicating adaptation to gallinaceous poultry, potentially extends that estimate before the first detections. The estimated tMRCA of LPAIV and HPAIV from the HPAIV-positive premises was March 19, 2020 (95% BCI March 11, 2020–March 28, 2020; PP = 0.99); however, these estimates are countered by serial weekly negative diagnostic tests obtained from the same flock before onset of clinical signs. Other indicators suggestive of early detection of LPAI and mutation to HPAI include HPAIV being identified in only 1 of the 5 houses, each of the HPAIV isolates demonstrating a mixed LPAIV/HPAIV population by next-generation sequencing; active virus replication (relative cycle threshold values for each of the houses: 37, 20, 18, 23, 29) with a low level of seropositivity (only 1 detectable H7 titer [1:32] of 10 serum samples collected in the HPAIV-affected house).

Although no wild bird–origin precursor had all 8 segments that corresponded to the North Carolina and South Carolina H7N3 viruses, the most probable progenitor gene was identified for each individual segment as LPAIV originating from wild waterfowl migrating along the Mississippi flyway (PP 0.92–1.00). These findings suggest that a precursor virus most likely emerged in wild waterfowl in the Mississippi flyway, with subsequent introduction into poultry via occasional virus spread between migratory flyways (*11*). The genomes of North American LPAIVs appear to reassort at a remarkably high rate with no apparent pattern of gene segment association (*12*).

Wild bird origin H7 LPAIVs have repeatedly spilled over from wild birds into poultry in North and South America, Europe, Asia, Africa, and Australia (*13*); on 28 recorded occasions, they have mutated into HPAIV (*1*). These findings highlight the importance of global surveillance in wild birds and continued vigilance in biosecurity and surveillance in worldwide poultry populations.

Appendix Additional information on highly pathogenic avian influenza A(H7N3) in poultry, United States.
