# Supplementary material for: Highly Pathogenic Avian Influenza A(H7N3) Virus in Poultry, United States, 2020
Source: Emerg Infect Dis. 2020 Dec;26(12):2966–9. doi: 10.3201/eid2612.202790 (PMC7706930; doi:10.3201/eid2612.202790)
Supplement: Appendix — Additional information on highly pathogenic avian influenza A(H7N3) in poultry, United States. [file 20-2790-Techapp-s1.pdf]

Article DOI: https://doi.org/10.3201/eid2612.202790

Highly Pathogenic Avian Influenza A(H7N3) Virus in Poultry, United States, 2020

Appendix

Materials and Methods

Virus Sampling and Sequencing

The initial detections were made through routine and active surveillance testing, and the enhanced surveillance of the affected and epidemiologically related premises followed. All the turkey farms that tested positive for low pathogenic avian influenza (LPAI) H7N3 recorded clinical signs related to avian influenza infection such as slight drops in egg production and mild respiratory signs. During the surveillance, influenza H7 was detected in another commercial turkey flock in South Carolina that reported an increased daily mortality (4.6%). Two samples from a single house at these premises was confirmed positive for highly pathogenic avian influenza (HPAI) H7N3 by sequencing; the other 4 houses in the same farm were confirmed positive for LPAI H7N3. In March and April, >3,700 pooled swabs from oropharynx and cloaca were tested, representing nearly 19,000 birds. Of those, 48 (1.3%) pooled samples from 13 premises tested positive for influenza A and 44 (1.2%) tested positive for H7 by gene PCR (*1*,*2*). A total of 29 H7N3 viruses were amplified by RT-PCR (*3*) and sequenced using the Ion Torrent Personal Genome Machine (Life Technologies, https://www.thermofisher.com) and/or MiSeq (Illumina, https://www.illumina.com) platforms, as previously described (*4*). De novo and directed assembly of genome sequences were carried out using the SeqMan NGen v. Four program. Nucleotide sequences for complete genomes of H7N3 viruses have been deposited in GenBank under accession nos. MT444183–MT444350 and MT444352–MT444415.

Phylogenetic Analysis

Genome sequences of wild bird avian influenza viruses (AIVs) close to the initial H7N3 viruses in turkeys were examined by BLAST function (https://blast.ncbi.nlm.nih.gov, accessed March 2020; 100 outputs), and added to the NC and SC H7N3 viruses analysis for each gene

segment. Alignment of the complete genome sequences was performed using MAFFT (http://mafft.cbrc.jp/alignment/software). Maximum-likelihood (ML) phylogeny of each gene segment was generated by using RAxML (*5*). Bootstrap support values were generated using 1,000 rapid replicates.

To estimate viral gene transition and demographic history of each segment, we inferred time-scaled phylogenies by Bayesian Markov chain Monte Carlo (MCMC) sampling using BEAST v1.10.4 (https://www.mybiosoftware.com). From the ML phylogenetic analysis, potential outliers that considerably deviated from the root-to-tip distance against time were removed from this study by using TempEst v1.5.1 (*6*). To trace the most probable ancestral wild bird viruses that generated the North Carolina and South Carolina H7N3 viruses, a discrete state ancestral reconstruction approach was implemented using bird flyways as discrete ancestral character states, including the Atlantic, Mississippi, Central, and Pacific flyways. We used an uncorrelated lognormal distribution relaxed clock method, the HKY nucleotide substitution model, and the GMRF Bayesian skyride coalescent prior (*7*). An asymmetric substitution model with the Bayesian stochastic search variable selection and a strict clock model were used for the discrete nominal category. At least 3 independent Markov chains were performed to meet the basis of the criterion of an effective sampling size of >200 as calculated by Tracer version 1.5 with a 10% burn-in (http://tree.bio.ed.ac.uk/software/tracer). Maximum clade credibility trees were generated for each gene segment by using TreeAnnotator (http://www. phylo.org/index.php/tools/treeannotator.html) and were visualized using FigTree 1.4.4 (http://tree.bio.ed.ac.uk).

To increase phylogenetic resolution, nucleotide sequences of the entire protein coding regions of each virus were concatenated with modified hemagglutinin (HA) and neuraminidase (NA) gene sequences to exclude the insertion at the HA cleavage site and the deletion at the NA stalk region, and then analyzed with Bayesian and median-joining (MJ) network phylogenetic analysis (*8*,*9*). Based on marginal likelihoods of various demographic models estimated by path sampling and stepping-stone analysis, we chose the relaxed clock model, the HKY nucleotide substitution model, and coalescent exponential population prior for a Bayesian framework (*10*). At least 3 independent Markov chains were performed to meet the basis of the criterion of an effective sampling size of >200 as calculated by Tracer version 1.5 with a 10% burn-in.

Maximum clade credibility trees were generated for each gene segment by using TreeAnnotator and were visualized by using FigTree 1.4.4.

Pathotyping Test

The intravenous pathogenicity index (IVPI) test was conducted in chickens according to the OIE Manual of Diagnostic Tests and Vaccines for Terrestrial Animals (http://www.oie.int/en/international-standard-setting/terrestrial-manual). In brief, 0.1 mL of infectious allantoic fluid was inoculated intravenously into 10 6-week-old specific-pathogen-free chickens and the chickens were monitored for 10 days for illness and death. Isolates with IVPI >1.2 were characterized as HPAIV. The challenge studies and all experiments with live viruses were conducted in a biosafety level 3 facility at the National Veterinary Services Laboratories, Animal and Plant Health Inspection Service, US Department of Agriculture in Ames, Iowa, USA, in accordance with approved institutional animal care and use protocols.

References

1. Spackman E. Avian influenza virus detection and quantitation by real-time RT-PCR. Methods Mol Biol. 2020;2123:137–48. PubMed https://doi.org/10.1007/978-1-0716-0346-8_11

2. Spackman E, Senne DA, Myers TJ, Bulaga LL, Garber LP, Perdue ML, et al. Development of a real-time reverse transcriptase PCR assay for type A influenza virus and the avian H5 and H7 hemagglutinin subtypes. J Clin Microbiol. 2002;40:3256–60. PubMed https://doi.org/10.1128/jcm.40.9.3256-3260.2002

3. Lee DH. Complete genome sequencing of influenza A viruses using next-generation sequencing. Methods Mol Biol. 2020;2123:69–79. PubMed https://doi.org/10.1007/978-1-0716-0346-8_6

4. Lee DH, Torchetti MK, Killian ML, Swayne DE. Deep sequencing of H7N8 avian influenza viruses from surveillance zone supports H7N8 high pathogenicity avian influenza was limited to a single outbreak farm in Indiana during 2016. Virology. 2017;507:216–9. PubMed https://doi.org/10.1016/j.virol.2017.04.025

5. Stamatakis A. RAxML version 8: a tool for phylogenetic analysis and post-analysis of large phylogenies. Bioinformatics. 2014;30:1312–3. PubMed https://doi.org/10.1093/bioinformatics/btu033

6. Rambaut A, Lam TT, Max Carvalho L, Pybus OG. Exploring the temporal structure of heterochronous sequences using TempEst (formerly Path-O-Gen). Virus Evol. 2016;2:vew007. https://doi.org/10.1093/ve/vew007

7. Minin VN, Bloomquist EW, Suchard MA. Smooth skyride through a rough skyline: Bayesian coalescent-based inference of population dynamics. Mol Biol Evol. 2008;25:1459–71. PubMed https://doi.org/10.1093/molbev/msn090

8. Bandelt HJ, Forster P, Röhl A. Median-joining networks for inferring intraspecific phylogenies. Mol Biol Evol. 1999;16:37–48. PubMed https://doi.org/10.1093/oxfordjournals.molbev.a026036

9. Suchard MA, Lemey P, Baele G, Ayres DL, Drummond AJ, Rambaut A. Bayesian phylogenetic and phylodynamic data integration using BEAST 1.10. Virus Evol. 2018;4:vey016. https://doi.org/10.1093/ve/vey016

10. Baele G, Li WL, Drummond AJ, Suchard MA, Lemey P. Accurate model selection of relaxed molecular clocks in Bayesian phylogenetics. Mol Biol Evol. 2013;30:239–43. PubMed https://doi.org/10.1093/molbev/mss243

Appendix Table 1. Avian influenza H7N3 viruses isolated and sequenced in this study*

Date collected	Virus strain	Premises	Operation type	Changes in HA cleavage site	Changes in NA stalk region	IVPI
2020 Mar 9	A/turkey/North_Carolina/20–007949–001/2020(H7N3)	NC-01	Commercial, breeder	N/A	N/A	0
2020 Mar 9	A/turkey/North_Carolina/20–007949–002/2020(H7N3)	NC-01	Commercial, breeder	N/A	N/A	ND
2020 Mar 10	A/turkey/North_Carolina/20–007948–002/2020(H7N3)	NC-04	Commercial, meat-type	N/A	N/A	ND
2020 Mar 10	A/turkey/North_Carolina/20–007948–003/2020(H7N3)	NC-04	Commercial, meat-type	N/A	N/A	ND
2020 Mar 10	A/turkey/North_Carolina/20–007948–005/2020(H7N3)	NC-04	Commercial, meat-type	N/A	N/A	ND
2020 Mar 12	A/turkey/North_Carolina/20–008256–001/2020(H7N3)	NC-01	Commercial, breeder	N/A	N/A	ND
2020 Mar 12	A/turkey/North_Carolina/20–008256–002/2020(H7N3)	NC-01	Commercial, breeder	N/A	N/A	ND
2020 Mar 12	A/turkey/North_Carolina/20–008257–001/2020(H7N3)	NC-03	Commercial, meat-type	Single basic amino acid substitution	N/A	0
2020 Mar 12	A/turkey/North_Carolina/20–008257–002/2020(H7N3)	NC-03	Commercial, meat-type	Single basic amino acid substitution	N/A	ND
2020 Mar 12	A/turkey/North_Carolina/20–008258–001/2020(H7N3)	NC-04	Commercial, meat-type	N/A	N/A	ND
2020 Mar 12	A/turkey/North_Carolina/20–008258–002/2020(H7N3)	NC-04	Commercial, meat-type	N/A	N/A	ND
2020 Mar 13	A/turkey/North_Carolina/20–008395–002/2020(H7N3)	NC-02	Commercial, meat-type	Single basic amino acid substitution	N/A	ND
2020 Mar 13	A/turkey/North_Carolina/20–008395–003/2020(H7N3)	NC-02	Commercial, meat-type	Single basic amino acid substitution	N/A	ND
2020 Mar 13	A/turkey/North_Carolina/20–008423–002/2020(H7N3)	NC-05	Commercial, meat-type	N/A	66 nt deletion	ND
2020 Mar 13	A/turkey/North_Carolina/20–008425–001/2020(H7N3)	NC-06	Commercial, meat-type	N/A	66 nt deletion	0
2020 Mar 13	A/turkey/South_Carolina/20–008394–001/2020(H7N3)	SC-01	Commercial, meat-type	N/A	N/A	0
2020 Mar 15	A/turkey/North_Carolina/20–008421–001/2020(H7N3)	NC-07	Commercial, meat-type	N/A	N/A	ND
2020 Mar 17	A/turkey/North_Carolina/20–008734–001/2020(H7N3)	NC-07	Commercial, breeder	N/A	N/A	ND
2020 Mar 19	A/turkey/North_Carolina/20–009000–001/2020(H7N3)	NC-08	Commercial, meat-type	N/A	N/A	ND
2020 Mar 19	A/turkey/North_Carolina/20–009000–002/2020(H7N3)	NC-08	Commercial, meat-type	N/A	N/A	ND
2020 Mar 19	A/turkey/North_Carolina/20–009000–004/2020(H7N3)	NC-08	Commercial, meat-type	N/A	N/A	ND
2020 Mar 30	A/turkey/North_Carolina/20–010036–002/2020(H7N3)	NC-09	Commercial, meat-type	N/A	N/A	ND
2020 Mar 30	A/turkey/North_Carolina/20–010036–004/2020(H7N3)	NC-09	Commercial, meat-type	N/A	N/A	ND
2020 Mar 30	A/turkey/North_Carolina/20–010036–005/2020(H7N3)	NC-09	Commercial, meat-type	N/A	N/A	ND
2020 Apr 6	A/turkey/South_Carolina/20–010561–002/2020(H7N3)	SC-02	Commercial, meat-type	N/A	N/A	0
2020 Apr 6	A/turkey/South_Carolina/20–010561–003/2020(H7N3)	SC-02	Commercial, meat-type	27 nt insertion	N/A	ND
2020 Apr 6	A/turkey/South_Carolina/20–010561–004/2020(H7N3)	SC-02	Commercial, meat-type	N/A	N/A	ND
2020 Apr 6	A/turkey/South_Carolina/20–010561–005/2020(H7N3)	SC-02	Commercial, meat-type	N/A	N/A	ND
2020 Apr 6	A/turkey/South_Carolina/20–010561–006/2020(H7N3)	SC-02	Commercial, meat-type	27 nt insertion	N/A	2.47

*HA, hemagglutinin; IVPI, intravenous pathogenicity index; NA, neuraminidase; N/A, not applicable; ND, not done.

Appendix Table 2. Mammalian host-specific genetic markers*

	Segment and amino acid change															
	HA		PB2			PB1		PA		NP			NS1			
Strain	Q226L	G228S	Q591K	E627K	D701N	K353R	T566A	S409N	K615N	R99K	N319K	S345N	S42P	D92E	F103L	M106I
A/turkey/North_Carolina/20–007948–002/2020	Q	G	Q	E	D	K	T	S	K	R	N	S	S	D	F	M
A/turkey/North_Carolina/20–007948–003/2020	Q	G	Q	E	D	K	T	S	K	R	N	S	S	D	F	M
A/turkey/North_Carolina/20–007948–005/2020	Q	G	Q	E	D	K	T	S	K	R	N	S	S	D	F	M
A/turkey/North_Carolina/20–007949–001/2020	Q	G	Q	E	D	K	T	S	K	R	N	S	S	D	F	M
A/turkey/North_Carolina/20–007949–002/2020	Q	G	Q	E	D	K	T	S	K	R	N	S	S	D	F	M
A/turkey/North_Carolina/20–008256–001/2020	Q	G	Q	E	D	K	T	S	K	R	N	S	S	D	F	M
A/turkey/North_Carolina/20–008256–002/2020	Q	G	Q	E	D	K	T	S	K	R	N	S	S	D	F	M
A/turkey/North_Carolina/20–008257–001/2020	Q	G	Q	E	D	K	T	S	K	R	N	S	S	D	F	M
A/turkey/North_Carolina/20–008257–002/2020	Q	G	Q	E	D	K	T	S	K	R	N	S	S	D	F	M
A/turkey/North_Carolina/20–008258–001/2020	Q	G	Q	E	D	K	T	S	K	R	N	S	S	D	F	M
A/turkey/North_Carolina/20–008258–002/2020	Q	G	Q	E	D	K	T	S	K	R	N	S	S	D	F	M
A/turkey/North_Carolina/20–008395–002/2020	Q	G	Q	E	D	K	T	S	K	R	N	S	S	D	F	M
A/turkey/North_Carolina/20–008395–003/2020	Q	G	Q	E	D	K	T	S	K	R	N	S	S	D	F	M
A/turkey/North_Carolina/20–008421–001/2020	Q	G	Q	E	D	K	T	S	K	R	N	S	S	D	F	M
A/turkey/North_Carolina/20–008423–002/2020	Q	G	Q	E	D	K	T	S	K	R	N	S	S	D	F	M
A/turkey/North_Carolina/20–008425–001/2020	Q	G	Q	E	D	K	T	S	K	R	N	S	S	D	F	M
A/turkey/North_Carolina/20–008734–001/2020	Q	G	Q	E	D	K	T	S	K	R	N	S	S	D	F	M
A/turkey/North_Carolina/20–009000–001/2020	Q	G	Q	E	D	K	T	S	K	R	N	S	S	D	F	M
A/turkey/North_Carolina/20–009000–002/2020	Q	G	Q	E	D	K	T	S	K	R	N	S	S	D	F	M
A/turkey/North_Carolina/20–009000–004/2020	Q	G	Q	E	D	K	T	S	K	R	N	S	S	D	F	M
A/turkey/North_Carolina/20–010036–002/2020	Q	G	Q	E	D	K	T	S	K	R	N	S	S	D	F	M
A/turkey/North_Carolina/20–010036–004/2020	Q	G	Q	E	D	K	T	S	K	R	N	S	S	D	F	M
A/turkey/North_Carolina/20–010036–005/2020	Q	G	Q	E	D	K	T	S	K	R	N	S	S	D	F	M

Strain	Segment and amino acid change															
	HA		PB2			PB1		PA		NP			NS1			
	Q226L	G228S	Q591K	E627K	D701N	K353R	T566A	S409N	K615N	R99K	N319K	S345N	S42P	D92E	F103L	M106I
A/turkey/South_Carolina/20–008394–001/2020	Q	G	Q	E	D	K	T	S	K	R	N	S	S	D	F	M
A/turkey/South_Carolina/20–010561–002/2020	Q	G	Q	E	D	K	T	S	K	R	N	S	S	D	F	M
A/turkey/South_Carolina/20–010561–003/2020	Q	G	Q	E	D	K	T	S	K	R	N	S	S	D	F	M
A/turkey/South_Carolina/20–010561–004/2020	Q	G	Q	E	D	K	T	S	K	R	N	S	S	D	F	M
A/turkey/South_Carolina/20–010561–005/2020	Q	G	Q	E	D	K	T	S	K	R	N	S	S	D	F	M
A/turkey/South_Carolina/20–010561–006/2020	Q	G	Q	E	D	K	T	S	K	R	N	S	S	D	F	M

* D, aspartic acid; E, glutaminc acid; F, phenylalanine; G, glycine; HA, hemagglutinin; K, lysine; M, methionine; N, asparagine; NP, nucleoprotein; NS, nonstructural protein; PA, polymerase; PB, polymerase basic; Q, glutamine; R, arginine; S, serine; T, threonine.

A

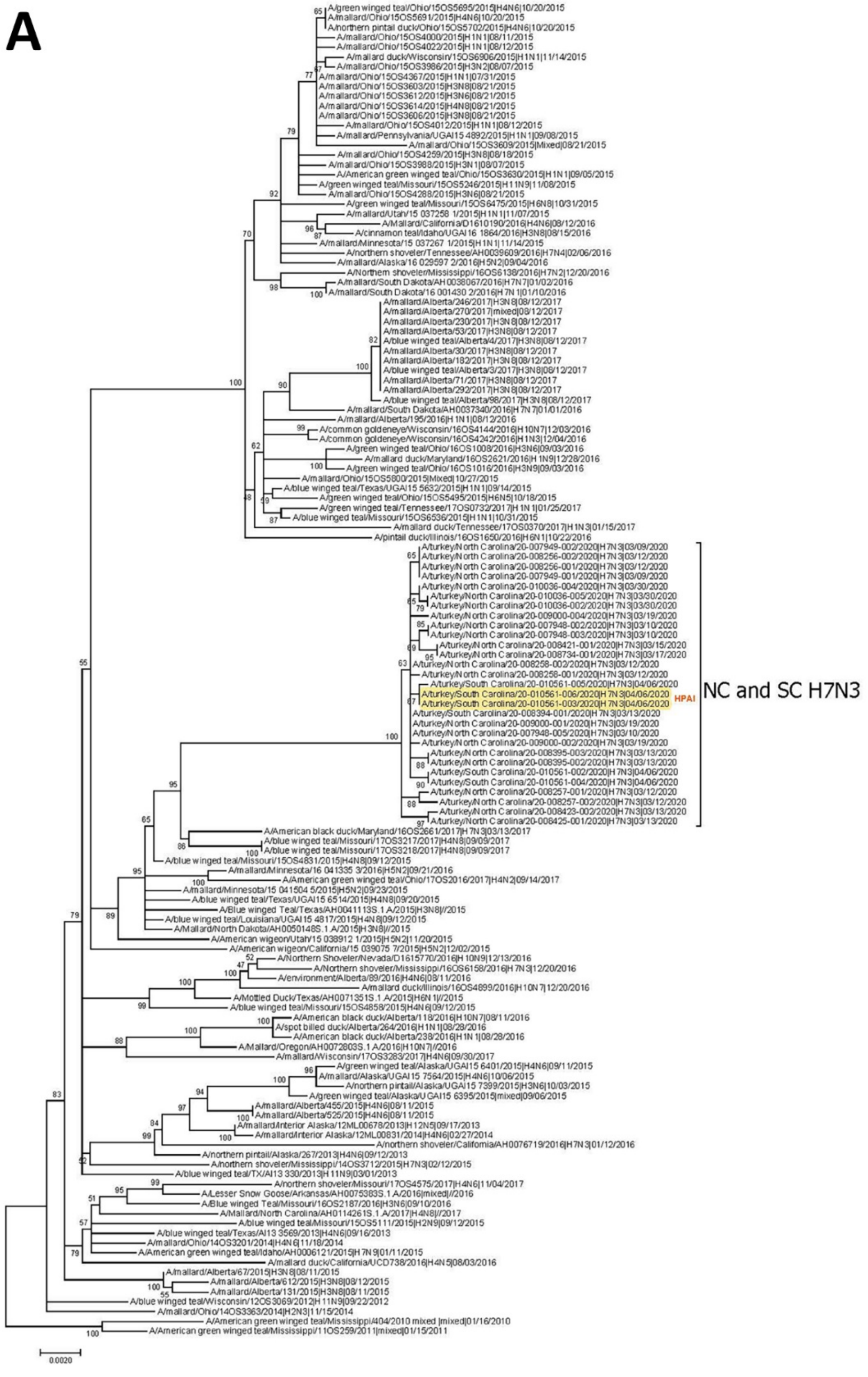

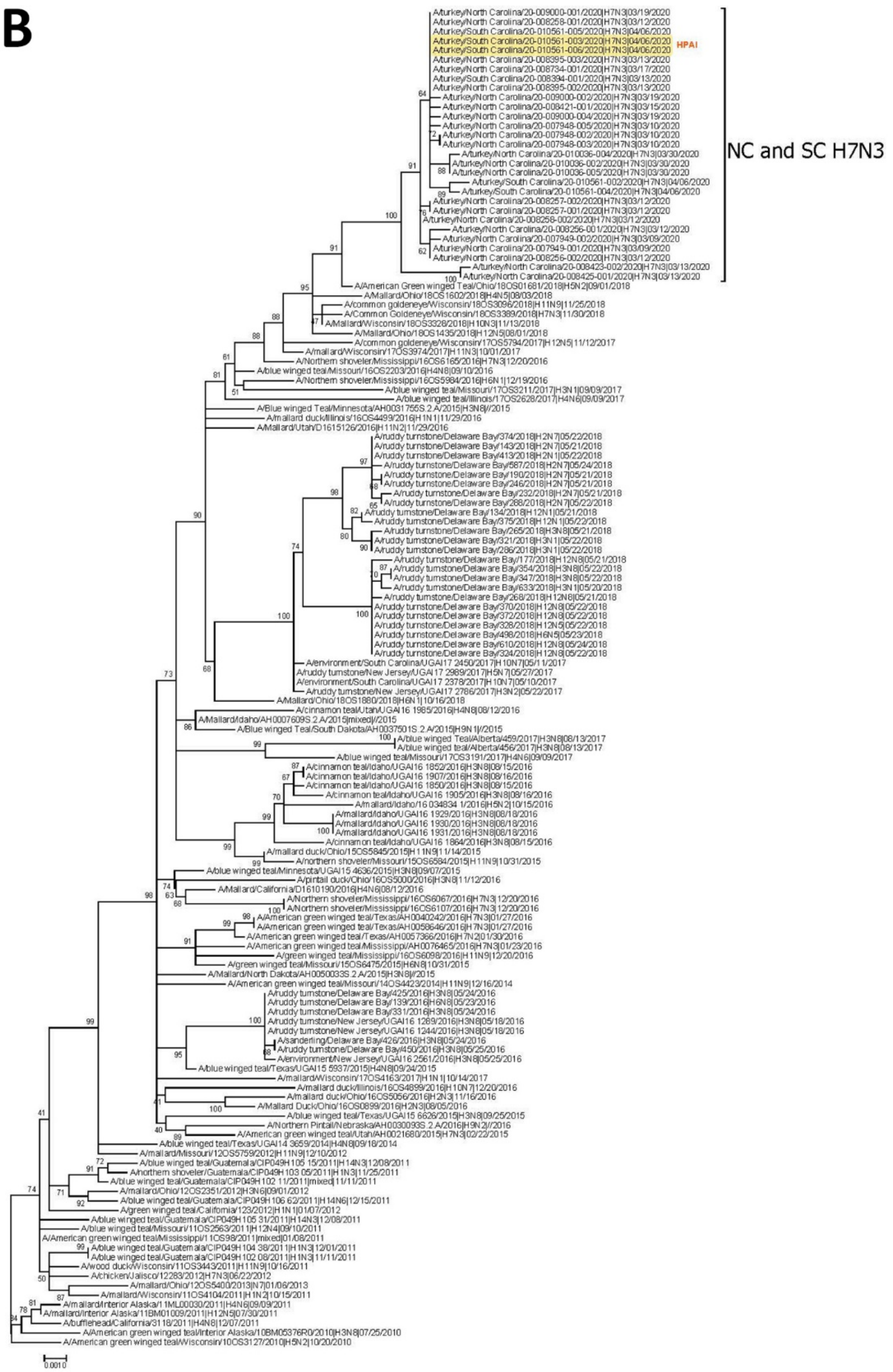
B
HPAI
NC and SC H7N3
0.0010

C

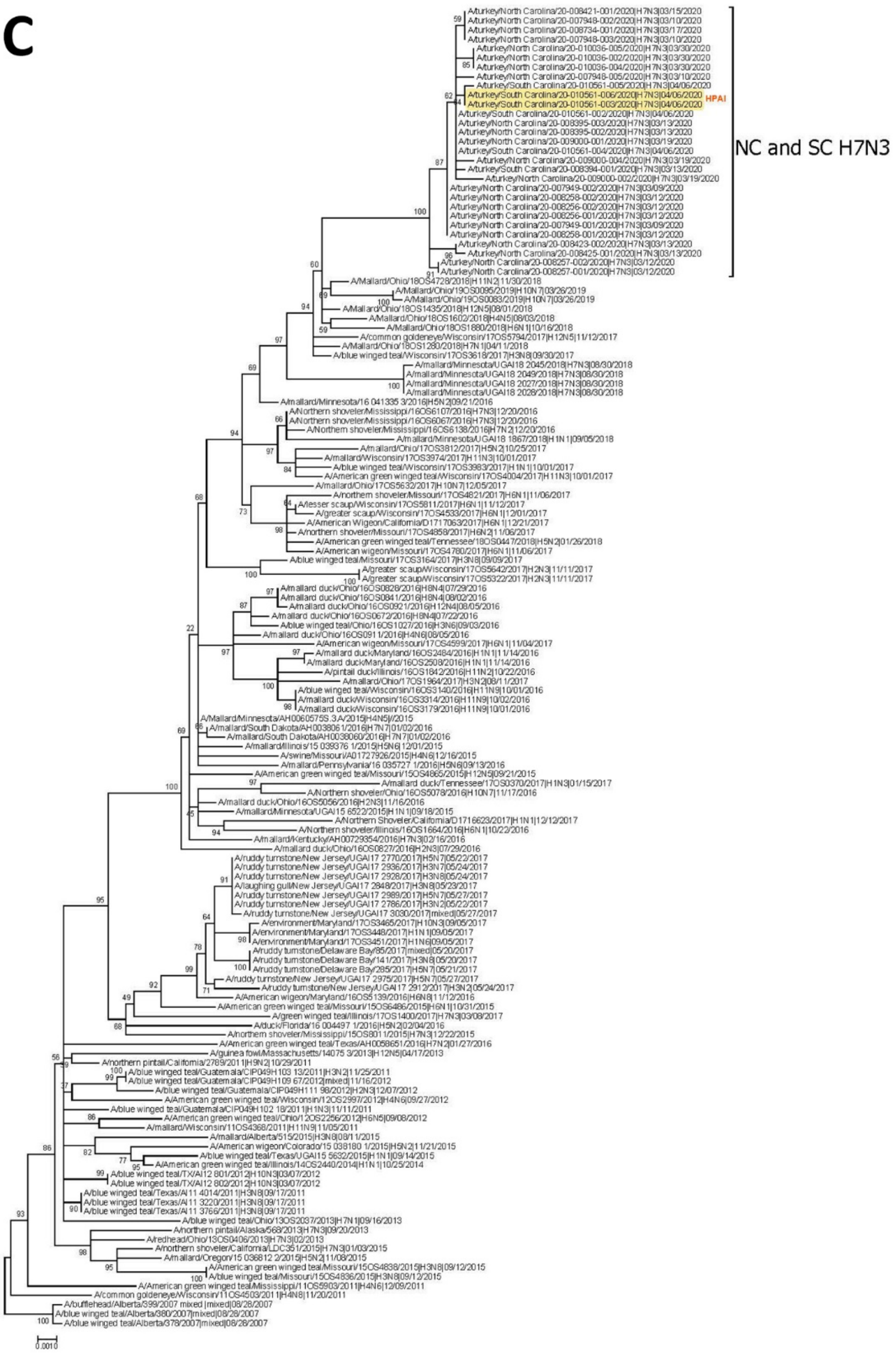

D

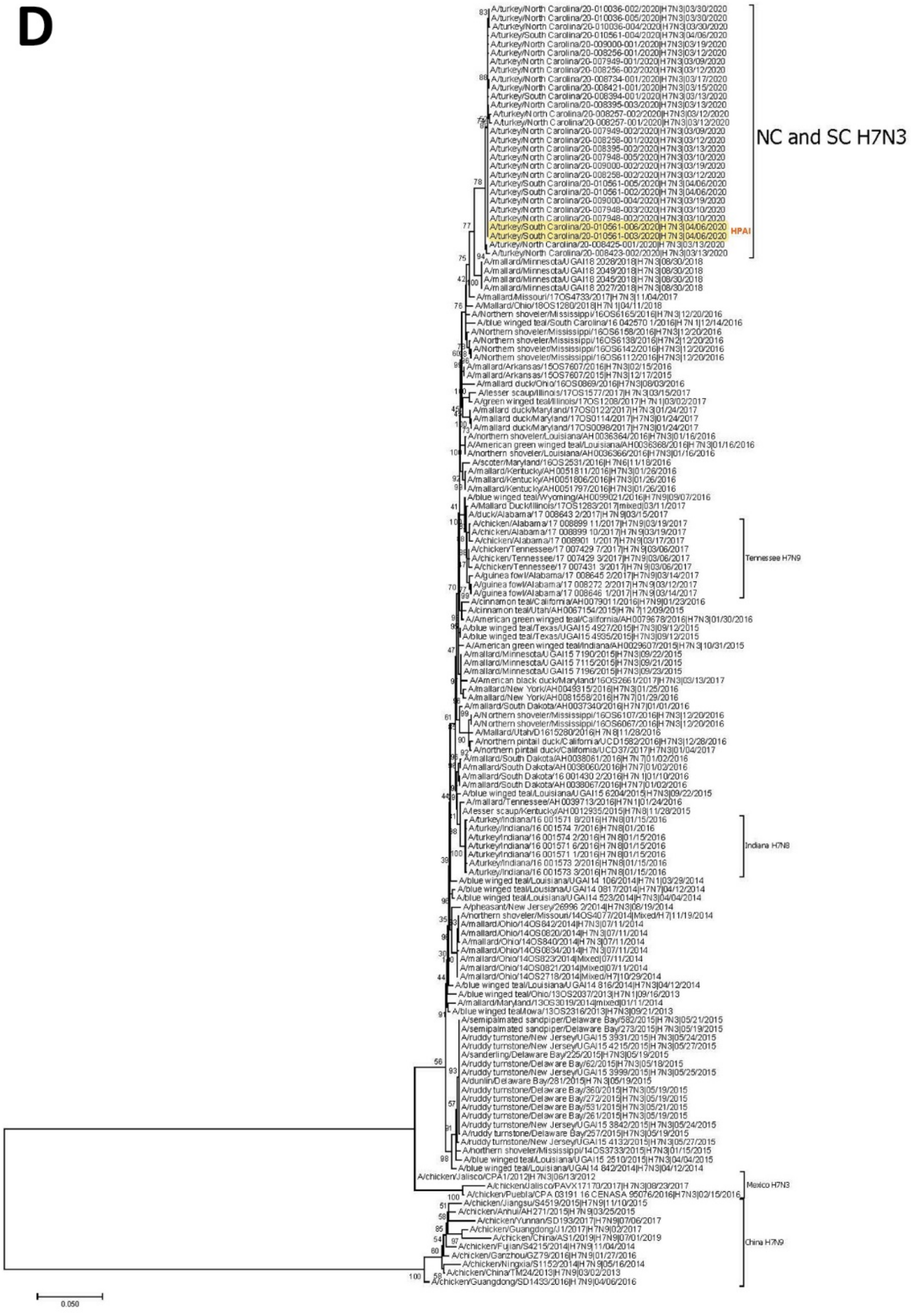

NC and SC H7N3
HPAI
Tennessee H7N9
Indiana H7N8
Mexico H7N3
China H7N9
0.050

E

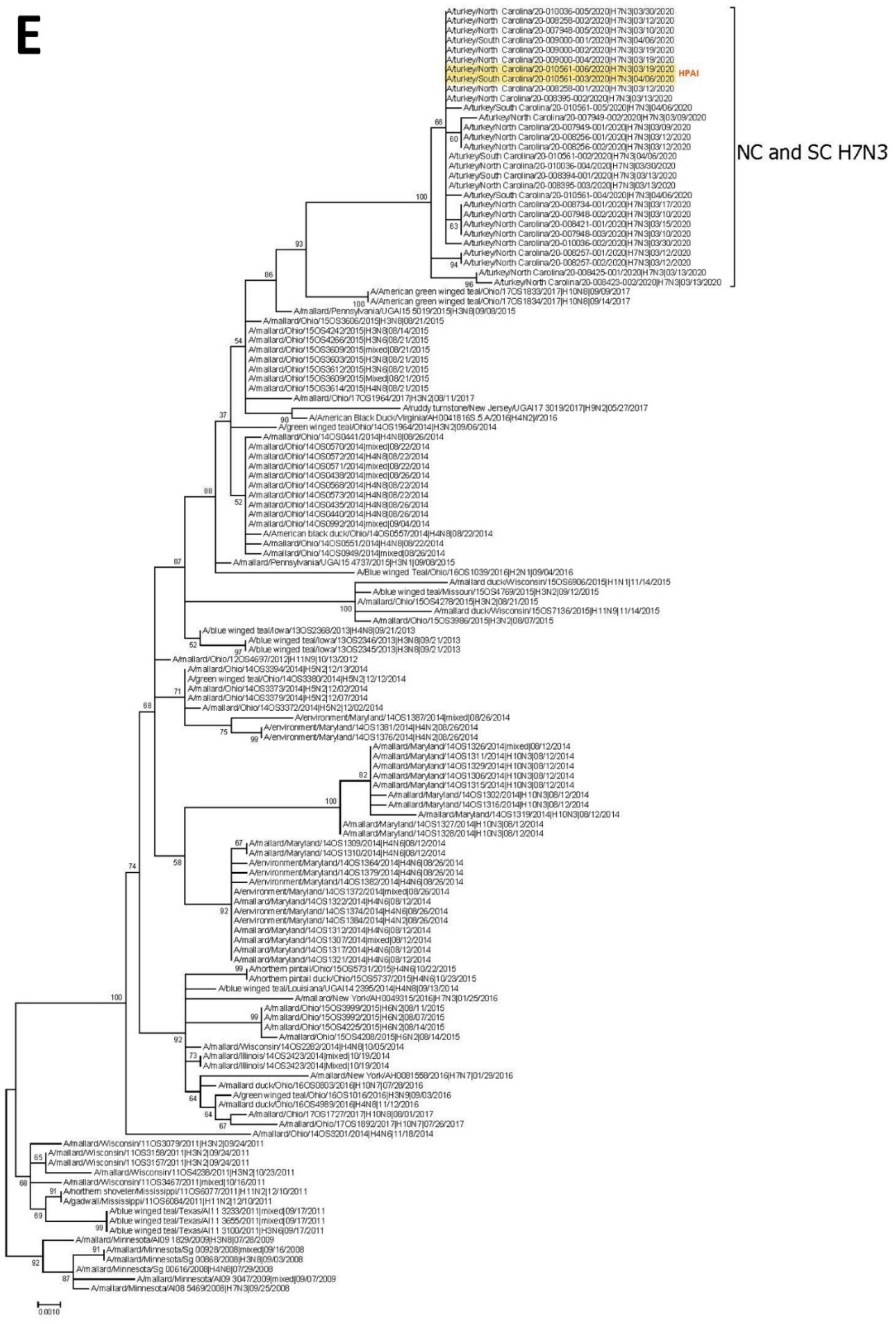

F

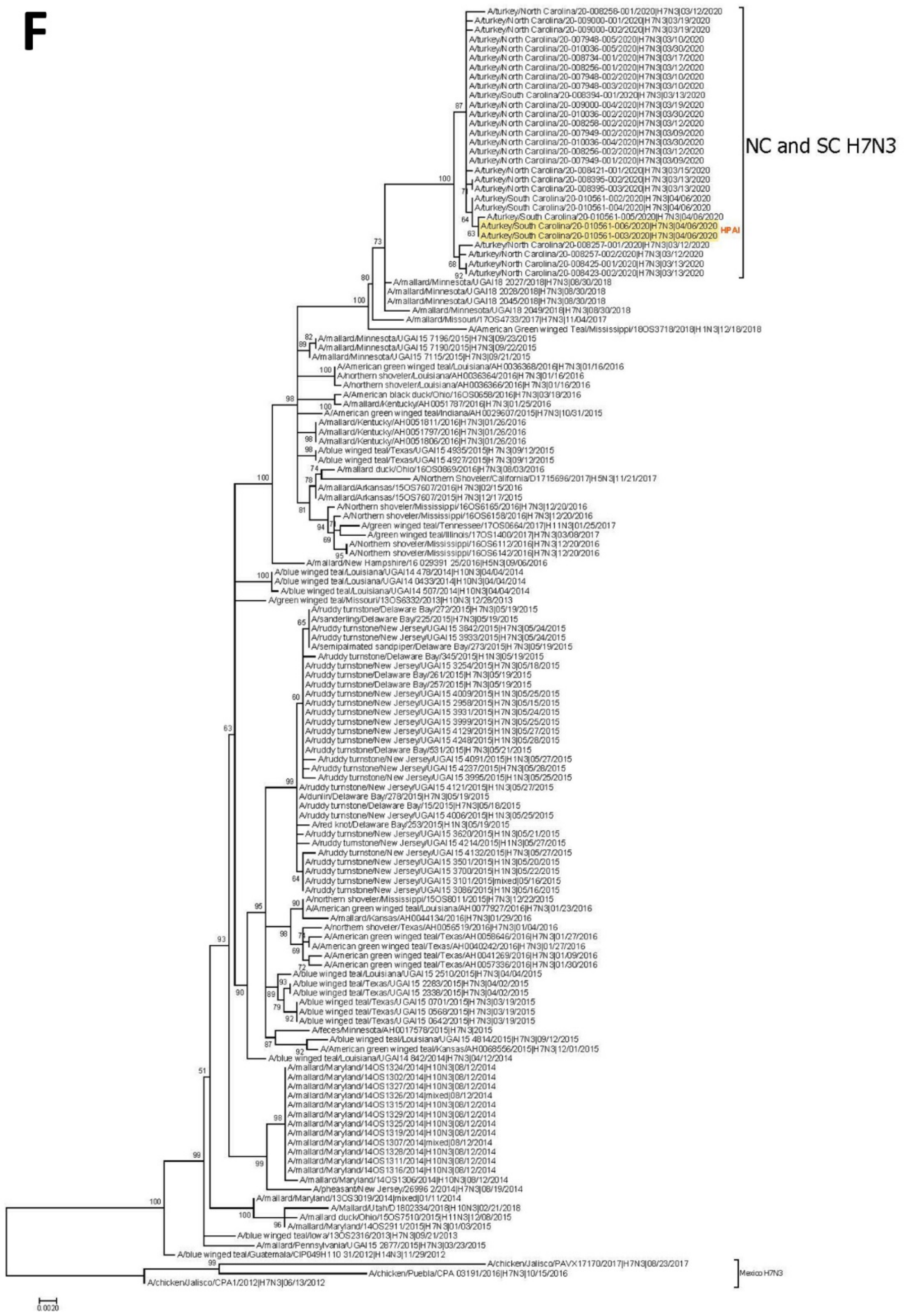

G

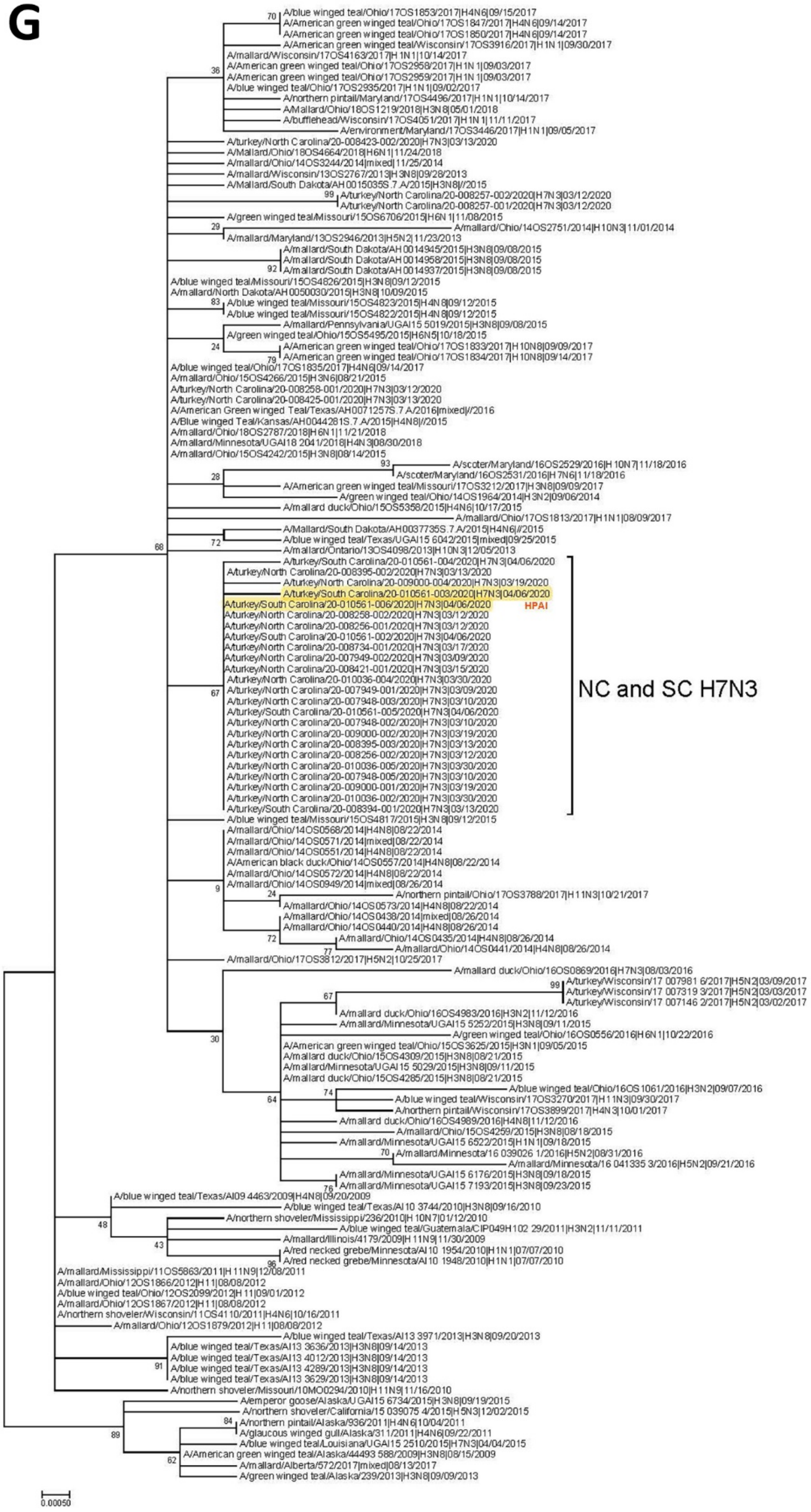

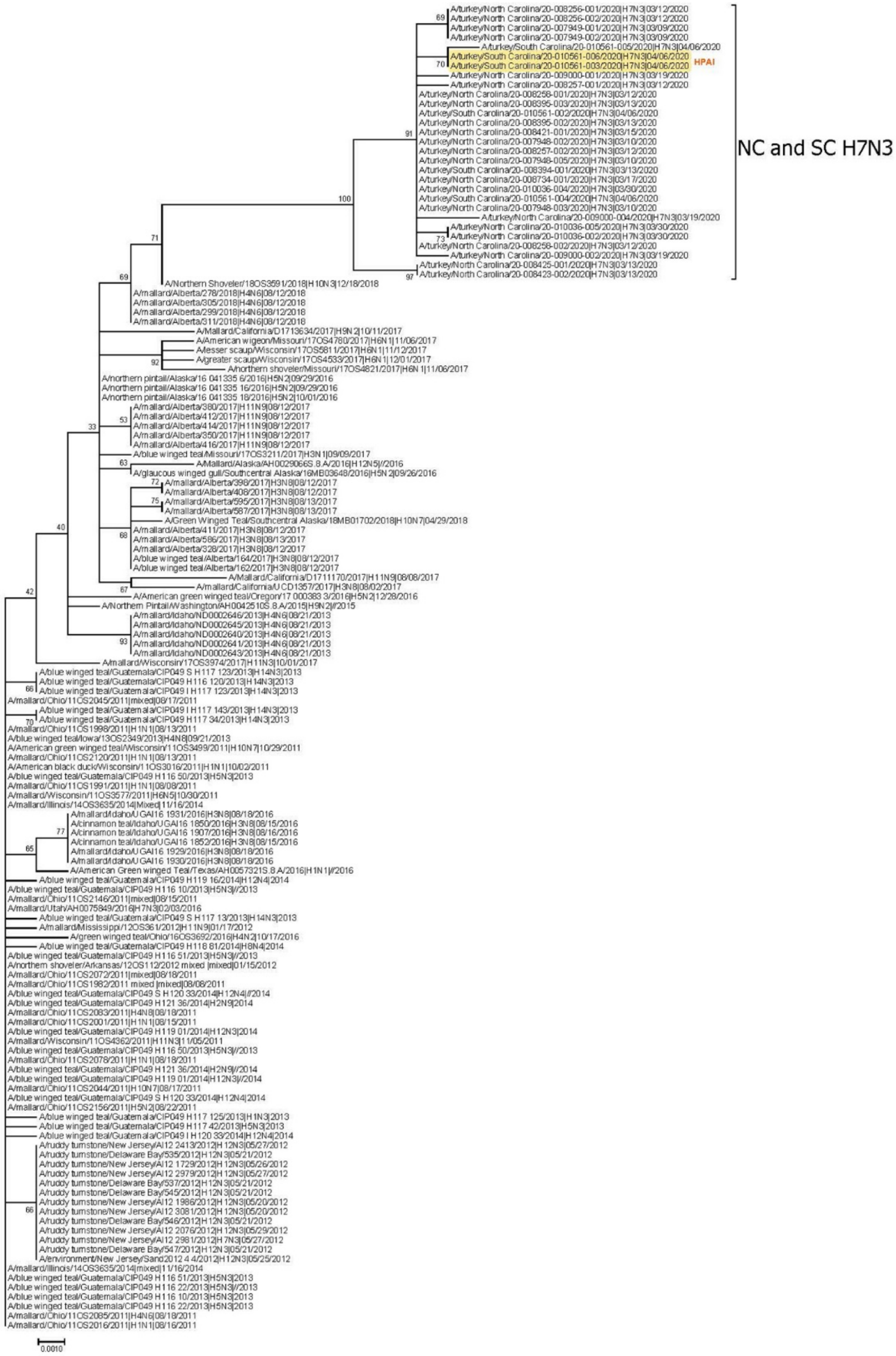

Appendix Figure 1. Maximum likelihood trees with ancestral wild bird avian influenza viruses. At each branch, the number indicates a bootstrap value. A) polymerase basic 2 (PB2); B) PB1; C) polymerase (PA); D) hemagglutinin (HA); E) nucleoprotein (NP); F) neuraminidase (NA); G) matrix (M); H) nonstructural (NS).

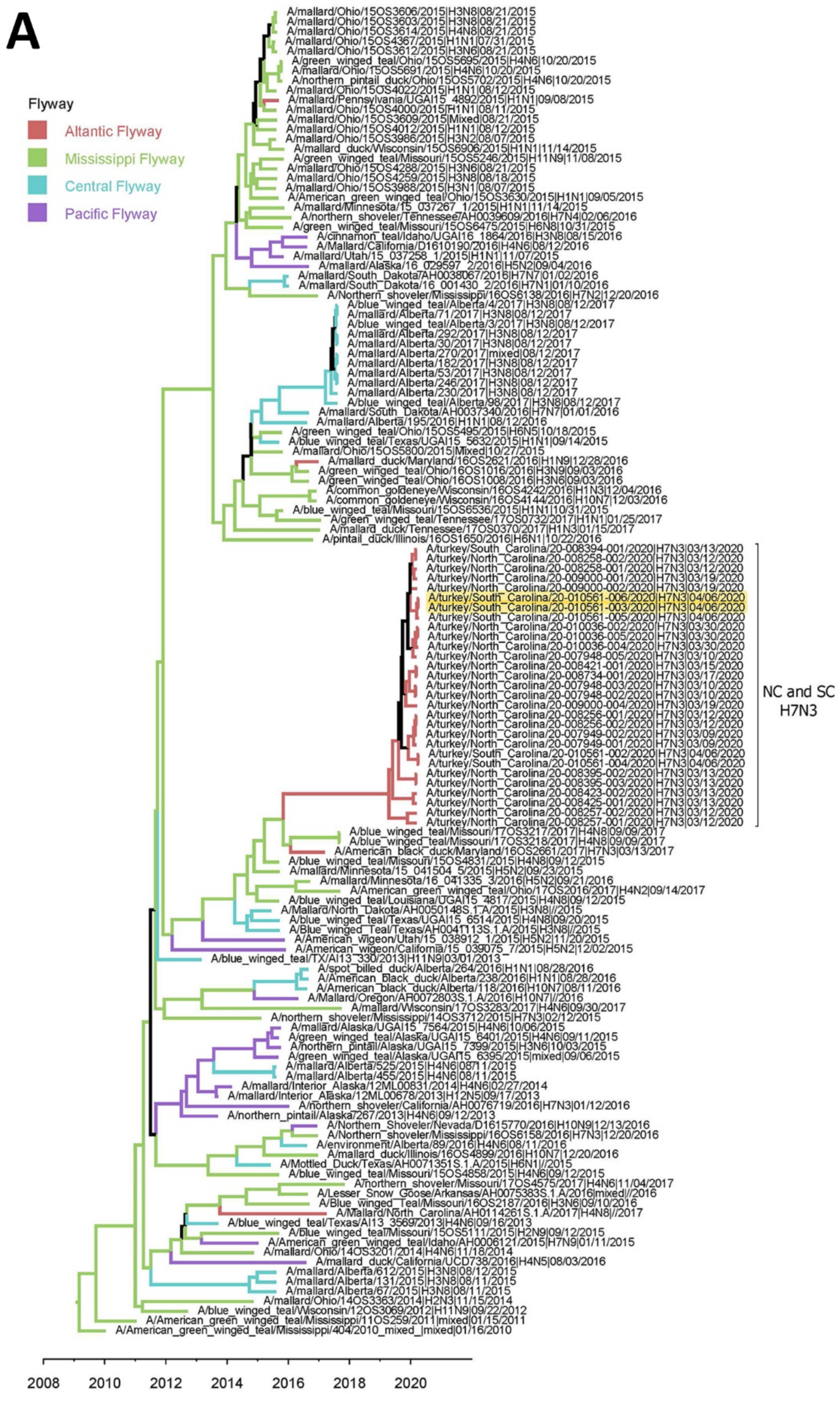
A
Flyway
Altantic Flyway
Mississippi Flyway
Central Flyway
Pacific Flyway
NC and SC H7N3
2008
2010
2012
2014
2016
2018
2020

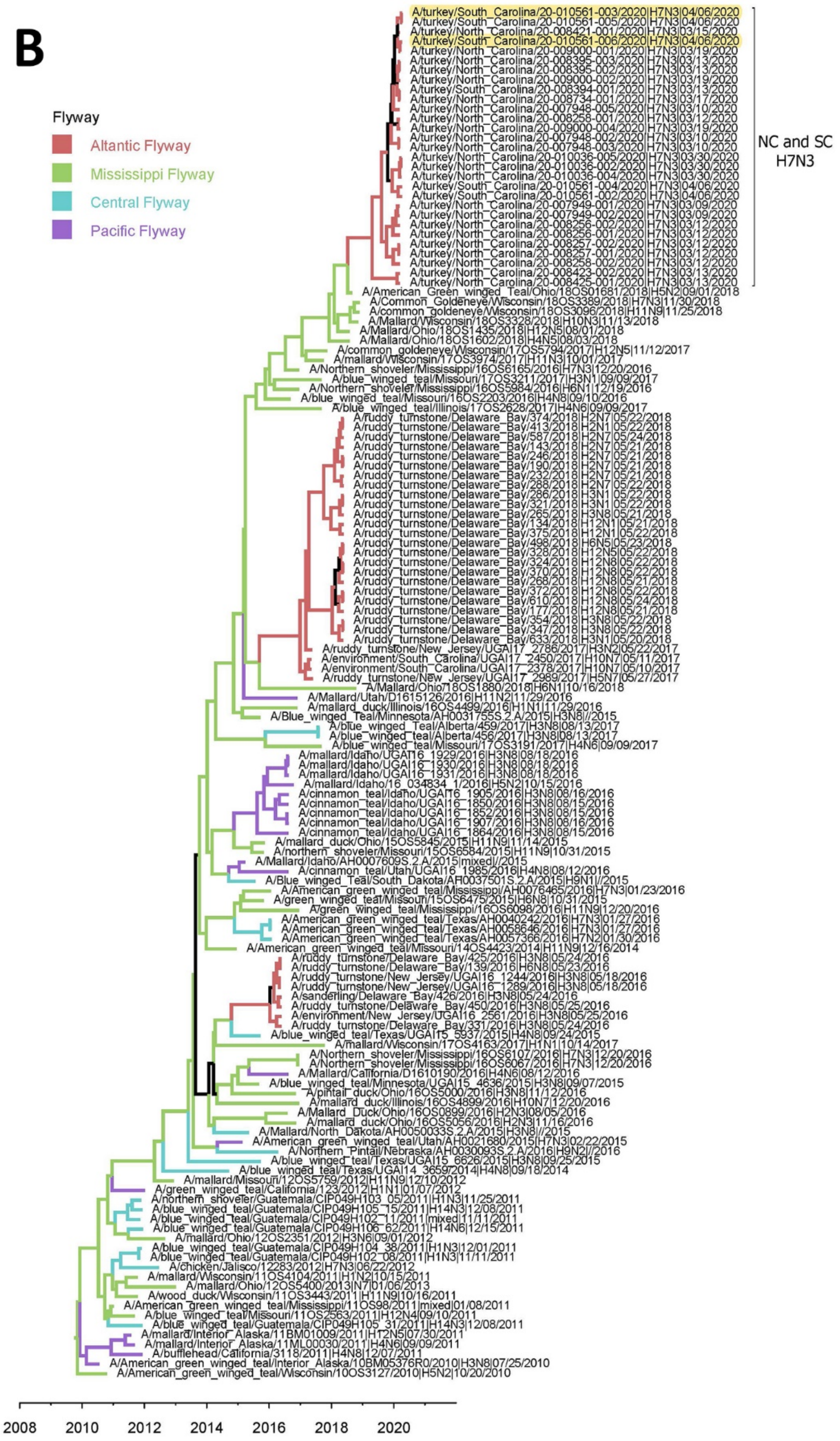
B
Flyway
Altantic Flyway
Mississippi Flyway
Central Flyway
Pacific Flyway
NC and SC
H7N3
2008
2010
2012
2014
2016
2018
2020

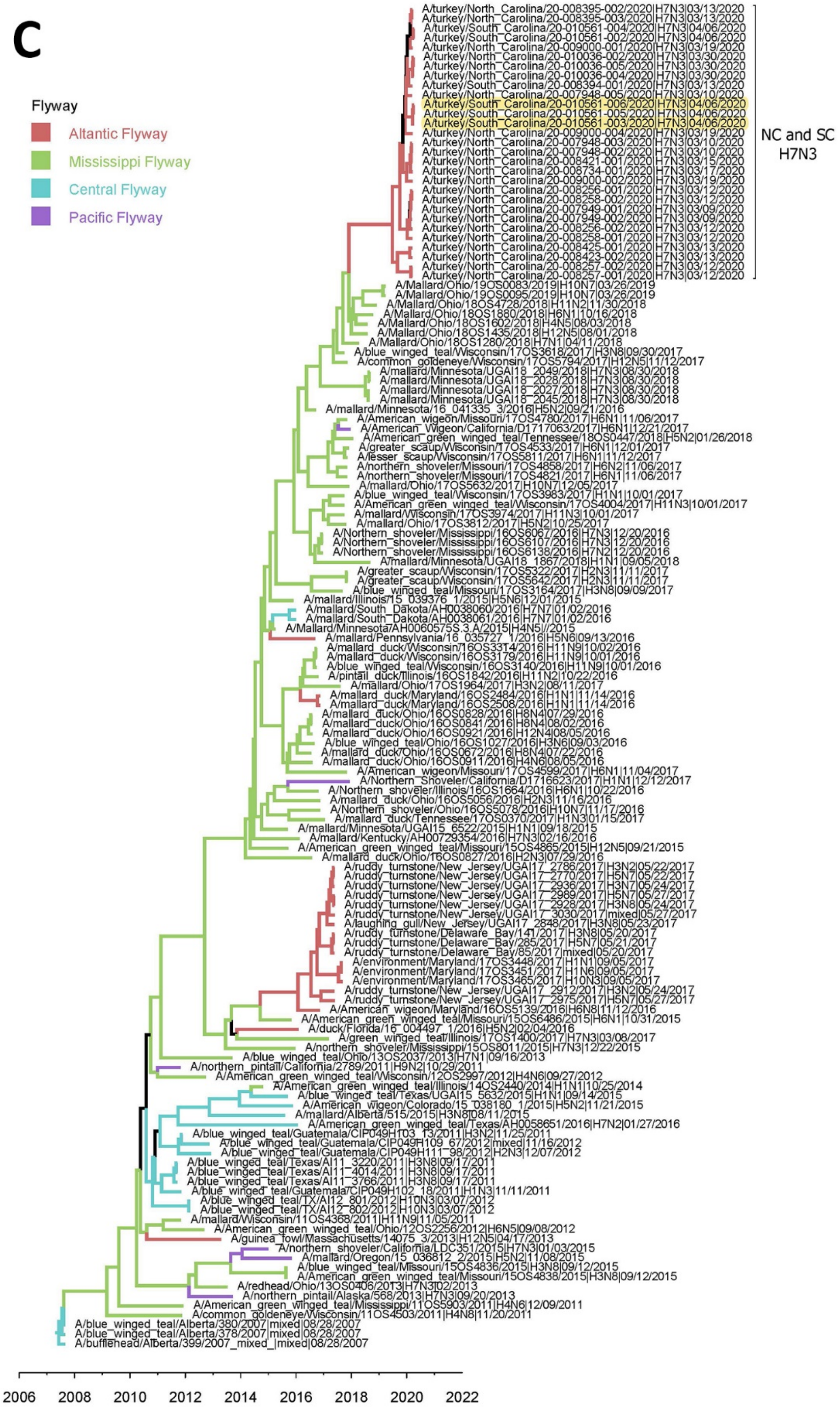
C
Flyway
Altantic Flyway
Mississippi Flyway
Central Flyway
Pacific Flyway
NC and SC
H7N3
2006
2008
2010
2012
2014
2016
2018
2020
2022

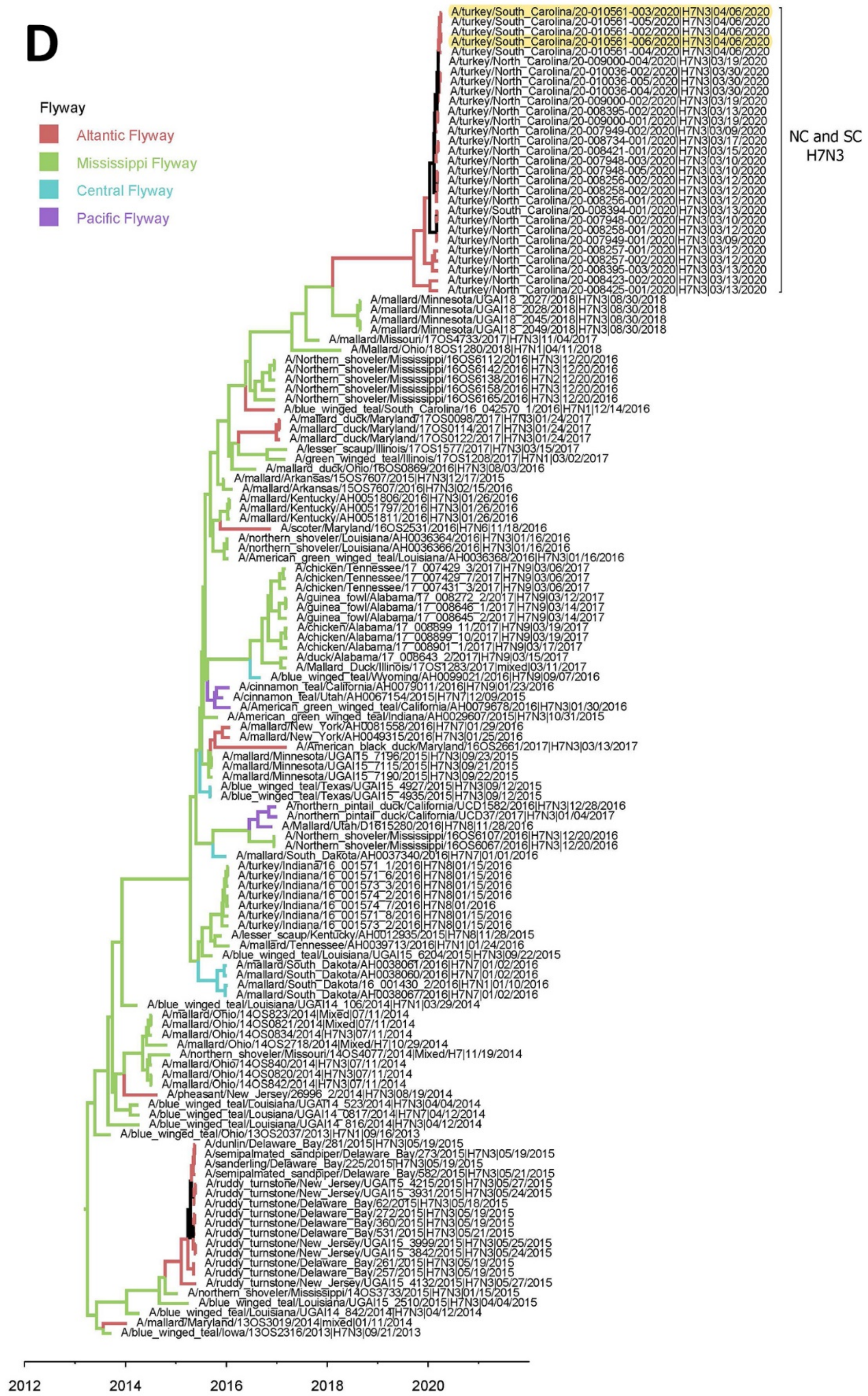
D
Flyway
Altantic Flyway
Mississippi Flyway
Central Flyway
Pacific Flyway
A/turkey/South_Carolina/20-010561-003/2020|H7N3|04/06/2020
A/turkey/South_Carolina/20-010561-005/2020|H7N3|04/06/2020
A/turkey/South_Carolina/20-010561-002/2020|H7N3|04/06/2020
A/turkey/South_Carolina/20-010561-006/2020|H7N3|04/06/2020
A/turkey/South_Carolina/20-010561-004/2020|H7N3|04/06/2020
A/turkey/North_Carolina/20-009000-004/2020|H7N3|03/19/2020
A/turkey/North_Carolina/20-010036-002/2020|H7N3|03/30/2020
A/turkey/North_Carolina/20-010036-005/2020|H7N3|03/30/2020
A/turkey/North_Carolina/20-010036-004/2020|H7N3|03/30/2020
A/turkey/North_Carolina/20-009000-002/2020|H7N3|03/19/2020
A/turkey/North_Carolina/20-008395-002/2020|H7N3|03/13/2020
A/turkey/North_Carolina/20-009000-001/2020|H7N3|03/19/2020
A/turkey/North_Carolina/20-007949-002/2020|H7N3|03/09/2020
A/turkey/North_Carolina/20-008734-001/2020|H7N3|03/17/2020
A/turkey/North_Carolina/20-008421-001/2020|H7N3|03/15/2020
A/turkey/North_Carolina/20-007948-003/2020|H7N3|03/10/2020
A/turkey/North_Carolina/20-007948-005/2020|H7N3|03/10/2020
A/turkey/North_Carolina/20-008256-002/2020|H7N3|03/12/2020
A/turkey/North_Carolina/20-008258-002/2020|H7N3|03/12/2020
A/turkey/North_Carolina/20-008256-001/2020|H7N3|03/12/2020
A/turkey/South_Carolina/20-008394-001/2020|H7N3|03/13/2020
A/turkey/North_Carolina/20-007948-002/2020|H7N3|03/10/2020
A/turkey/North_Carolina/20-008258-001/2020|H7N3|03/12/2020
A/turkey/North_Carolina/20-007949-001/2020|H7N3|03/09/2020
A/turkey/North_Carolina/20-008257-001/2020|H7N3|03/12/2020
A/turkey/North_Carolina/20-008257-002/2020|H7N3|03/12/2020
A/turkey/North_Carolina/20-008395-003/2020|H7N3|03/13/2020
A/turkey/North_Carolina/20-008423-002/2020|H7N3|03/13/2020
A/turkey/North_Carolina/20-008425-001/2020|H7N3|03/13/2020
NC and SC
H7N3
A/mallard/Minnesota/UGAI18_2027/2018|H7N3|08/30/2018
A/mallard/Minnesota/UGAI18_2028/2018|H7N3|08/30/2018
A/mallard/Minnesota/UGAI18_2045/2018|H7N3|08/30/2018
A/mallard/Minnesota/UGAI18_2049/2018|H7N3|08/30/2018
A/mallard/Missouri/17OS4733/2017|H7N3|11/04/2017
A/Mallard/Ohio/18OS1280/2018|H7N1|04/11/2018
A/Northern_shoveler/Mississippi/16OS6112/2016|H7N3|12/20/2016
A/Northern_shoveler/Mississippi/16OS6142/2016|H7N3|12/20/2016
A/Northern_shoveler/Mississippi/16OS6138/2016|H7N2|12/20/2016
A/Northern_shoveler/Mississippi/16OS6158/2016|H7N3|12/20/2016
A/Northern_shoveler/Mississippi/16OS6165/2016|H7N3|12/20/2016
A/blue_winged_teal/South_Carolina/16_042570_1/2016|H7N1|12/14/2016
A/mallard_duck/Maryland/17OS0098/2017|H7N3|01/24/2017
A/mallard_duck/Maryland/17OS0114/2017|H7N3|01/24/2017
A/mallard_duck/Maryland/17OS0122/2017|H7N3|01/24/2017
A/lesser_scaup/Illinois/17OS1577/2017|H7N3|03/15/2017
A/green_winged_teal/Illinois/17OS1208/2017|H7N1|03/02/2017
A/mallard_duck/Ohio/16OS0869/2016|H7N3|08/03/2016
A/mallard/Arkansas/15OS7607/2015|H7N3|12/17/2015
A/mallard/Arkansas/15OS7607/2016|H7N3|02/15/2016
A/mallard/Kentucky/AH0051806/2016|H7N3|01/26/2016
A/mallard/Kentucky/AH0051797/2016|H7N3|01/26/2016
A/mallard/Kentucky/AH0051811/2016|H7N3|01/26/2016
A/scoter/Maryland/16OS2531/2016|H7N6|11/18/2016
A/northern_shoveler/Louisiana/AH0036364/2016|H7N3|01/16/2016
A/northern_shoveler/Louisiana/AH0036366/2016|H7N3|01/16/2016
A/American_green_winged_teal/Louisiana/AH0036368/2016|H7N3|01/16/2016
A/chicken/Tennessee/17_007429_3/2017|H7N9|03/06/2017
A/chicken/Tennessee/17_007429_7/2017|H7N9|03/06/2017
A/chicken/Tennessee/17_007431_3/2017|H7N9|03/06/2017
A/guinea_fowl/Alabama/17_008272_2/2017|H7N9|03/12/2017
A/guinea_fowl/Alabama/17_008646_1/2017|H7N9|03/14/2017
A/guinea_fowl/Alabama/17_008645_2/2017|H7N9|03/14/2017
A/chicken/Alabama/17_008899_11/2017|H7N9|03/19/2017
A/chicken/Alabama/17_008899_10/2017|H7N9|03/19/2017
A/chicken/Alabama/17_008901_1/2017|H7N9|03/17/2017
A/duck/Alabama/17_008643_2/2017|H7N9|03/15/2017
A/Mallard_Duck/Illinois/17OS1283/2017|mixed|03/11/2017
A/blue_winged_teal/Wyoming/AH0099021/2016|H7N9|09/07/2016
A/cinnamon_teal/California/AH0079011/2016|H7N9|01/23/2016
A/cinnamon_teal/Utah/AH0067154/2015|H7N7|12/09/2015
A/American_green_winged_teal/California/AH0079678/2016|H7N3|01/30/2016
A/American_green_winged_teal/Indiana/AH0029607/2015|H7N3|10/31/2015
A/mallard/New_York/AH0081558/2016|H7N7|01/29/2016
A/mallard/New_York/AH0049315/2016|H7N3|01/25/2016
A/American_black_duck/Maryland/16OS2661/2017|H7N3|03/13/2017
A/mallard/Minnesota/UGAI15_7196/2015|H7N3|09/23/2015
A/mallard/Minnesota/UGAI15_7115/2015|H7N3|09/21/2015
A/mallard/Minnesota/UGAI15_7190/2015|H7N3|09/22/2015
A/blue_winged_teal/Texas/UGAI15_4927/2015|H7N3|09/12/2015
A/blue_winged_teal/Texas/UGAI15_4935/2015|H7N3|09/12/2015
A/northern_pintail_duck/California/UCD1582/2016|H7N3|12/28/2016
A/northern_pintail_duck/California/UCD37/2017|H7N3|01/04/2017
A/Mallard/Utah/D1615280/2016|H7N8|11/28/2016
A/Northern_shoveler/Mississippi/16OS6107/2016|H7N3|12/20/2016
A/Northern_shoveler/Mississippi/16OS6067/2016|H7N3|12/20/2016
A/mallard/South_Dakota/AH0037340/2016|H7N7|01/01/2016
A/turkey/Indiana/16_001571_1/2016|H7N8|01/15/2016
A/turkey/Indiana/16_001571_6/2016|H7N8|01/15/2016
A/turkey/Indiana/16_001573_3/2016|H7N8|01/15/2016
A/turkey/Indiana/16_001574_2/2016|H7N8|01/15/2016
A/turkey/Indiana/16_001574_7/2016|H7N8|01/2016
A/turkey/Indiana/16_001571_8/2016|H7N8|01/15/2016
A/turkey/Indiana/16_001573_2/2016|H7N8|01/15/2016
A/lesser_scaup/Kentucky/AH0012935/2015|H7N8|11/28/2015
A/mallard/Tennessee/AH0039713/2016|H7N1|01/24/2016
A/blue_winged_teal/Louisiana/UGAI15_6204/2015|H7N3|09/22/2015
A/mallard/South_Dakota/AH0038061/2016|H7N7|01/02/2016
A/mallard/South_Dakota/AH0038060/2016|H7N7|01/02/2016
A/mallard/South_Dakota/16_001430_2/2016|H7N1|01/10/2016
A/mallard/South_Dakota/AH0038067/2016|H7N7|01/02/2016
A/blue_winged_teal/Louisiana/UGAI14_106/2014|H7N1|03/29/2014
A/mallard/Ohio/14OS823/2014|Mixed|07/11/2014
A/mallard/Ohio/14OS0821/2014|Mixed|07/11/2014
A/mallard/Ohio/14OS0834/2014|H7N3|07/11/2014
A/mallard/Ohio/14OS2718/2014|Mixed/H7|10/29/2014
A/northern_shoveler/Missouri/14OS4077/2014|Mixed/H7|11/19/2014
A/mallard/Ohio/14OS840/2014|H7N3|07/11/2014
A/mallard/Ohio/14OS0820/2014|H7N3|07/11/2014
A/mallard/Ohio/14OS842/2014|H7N3|07/11/2014
A/pheasant/New_Jersey/26996_2/2014|H7N3|08/19/2014
A/blue_winged_teal/Louisiana/UGAI14_523/2014|H7N3|04/04/2014
A/blue_winged_teal/Lousiana/UGAI14_0817/2014|H7N7|04/12/2014
A/blue_winged_teal/Louisiana/UGAI14_816/2014|H7N3|04/12/2014
A/blue_winged_teal/Ohio/13OS2037/2013|H7N1|09/16/2013
A/dunlin/Delaware_Bay/281/2015|H7N3|05/19/2015
A/semipalmated_sandpiper/Delaware_Bay/273/2015|H7N3|05/19/2015
A/sanderling/Delaware_Bay/225/2015|H7N3|05/19/2015
A/semipalmated_sandpiper/Delaware_Bay/582/2015|H7N3|05/21/2015
A/ruddy_turnstone/New_Jersey/UGAI15_4215/2015|H7N3|05/27/2015
A/ruddy_turnstone/New_Jersey/UGAI15_3931/2015|H7N3|05/24/2015
A/ruddy_turnstone/Delaware_Bay/62/2015|H7N3|05/18/2015
A/ruddy_turnstone/Delaware_Bay/272/2015|H7N3|05/19/2015
A/ruddy_turnstone/Delaware_Bay/360/2015|H7N3|05/19/2015
A/ruddy_turnstone/Delaware_Bay/531/2015|H7N3|05/21/2015
A/ruddy_turnstone/New_Jersey/UGAI15_3999/2015|H7N3|05/25/2015
A/ruddy_turnstone/New_Jersey/UGAI15_3842/2015|H7N3|05/24/2015
A/ruddy_turnstone/Delaware_Bay/261/2015|H7N3|05/19/2015
A/ruddy_turnstone/Delaware_Bay/257/2015|H7N3|05/19/2015
A/ruddy_turnstone/New_Jersey/UGAI15_4132/2015|H7N3|05/27/2015
A/northern_shoveler/Mississippi/14OS3733/2015|H7N3|01/15/2015
A/blue_winged_teal/Louisiana/UGAI15_2510/2015|H7N3|04/04/2015
A/blue_winged_teal/Louisiana/UGAI14_842/2014|H7N3|04/12/2014
A/mallard/Maryland/13OS3019/2014|mixed|01/11/2014
A/blue_winged_teal/Iowa/13OS2316/2013|H7N3|09/21/2013
2012
2014
2016
2018
2020

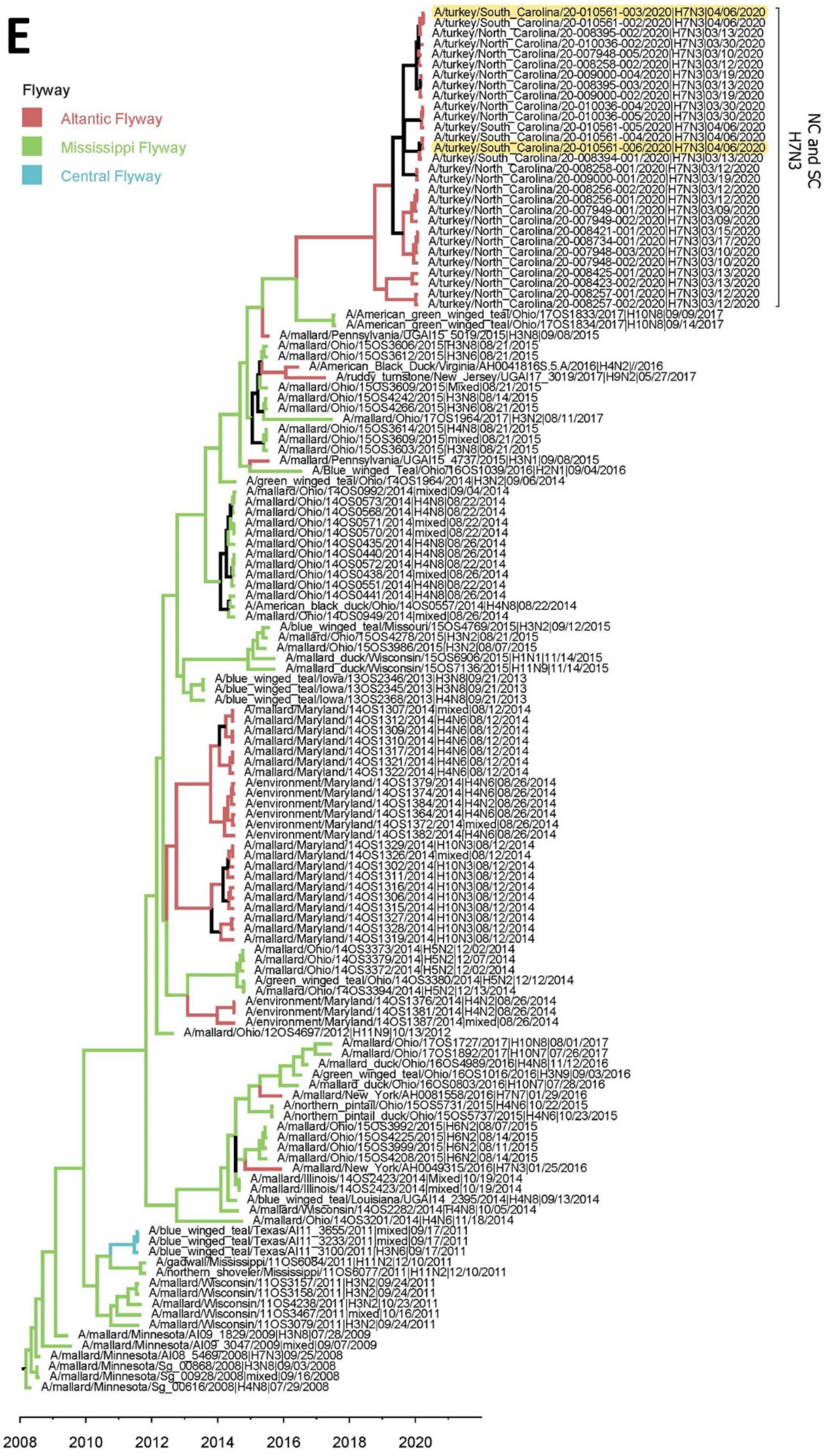
E
Flyway
Altantic Flyway
Mississippi Flyway
Central Flyway
A/turkey/South_Carolina/20-010561-003/2020|H7N3|04/06/2020
A/turkey/South_Carolina/20-010561-002/2020|H7N3|04/06/2020
A/turkey/North_Carolina/20-008395-002/2020|H7N3|03/13/2020
A/turkey/North_Carolina/20-010036-002/2020|H7N3|03/30/2020
A/turkey/North_Carolina/20-007948-005/2020|H7N3|03/10/2020
A/turkey/North_Carolina/20-008258-002/2020|H7N3|03/12/2020
A/turkey/North_Carolina/20-009000-004/2020|H7N3|03/19/2020
A/turkey/North_Carolina/20-008395-003/2020|H7N3|03/13/2020
A/turkey/North_Carolina/20-009000-002/2020|H7N3|03/19/2020
A/turkey/North_Carolina/20-010036-004/2020|H7N3|03/30/2020
A/turkey/North_Carolina/20-010036-005/2020|H7N3|03/30/2020
A/turkey/South_Carolina/20-010561-005/2020|H7N3|04/06/2020
A/turkey/South_Carolina/20-010561-004/2020|H7N3|04/06/2020
A/turkey/South_Carolina/20-010561-006/2020|H7N3|04/06/2020
A/turkey/South_Carolina/20-008394-001/2020|H7N3|03/13/2020
A/turkey/North_Carolina/20-008258-001/2020|H7N3|03/12/2020
A/turkey/North_Carolina/20-009000-001/2020|H7N3|03/19/2020
A/turkey/North_Carolina/20-008256-002/2020|H7N3|03/12/2020
A/turkey/North_Carolina/20-008256-001/2020|H7N3|03/12/2020
A/turkey/North_Carolina/20-007949-001/2020|H7N3|03/09/2020
A/turkey/North_Carolina/20-007949-002/2020|H7N3|03/09/2020
A/turkey/North_Carolina/20-008421-001/2020|H7N3|03/15/2020
A/turkey/North_Carolina/20-008734-001/2020|H7N3|03/17/2020
A/turkey/North_Carolina/20-007948-003/2020|H7N3|03/10/2020
A/turkey/North_Carolina/20-007948-002/2020|H7N3|03/10/2020
A/turkey/North_Carolina/20-008425-001/2020|H7N3|03/13/2020
A/turkey/North_Carolina/20-008423-002/2020|H7N3|03/13/2020
A/turkey/North_Carolina/20-008257-001/2020|H7N3|03/12/2020
A/turkey/North_Carolina/20-008257-002/2020|H7N3|03/12/2020
NC and SC
H7N3
A/American_green_winged_teal/Ohio/17OS1833/2017|H10N8|09/09/2017
A/American_green_winged_teal/Ohio/17OS1834/2017|H10N8|09/14/2017
A/mallard/Pennsylvania/UGAI15_5019/2015|H3N8|09/08/2015
A/mallard/Ohio/15OS3606/2015|H3N8|08/21/2015
A/mallard/Ohio/15OS3612/2015|H3N6|08/21/2015
A/American_Black_Duck/Virginia/AH0041816S.5.A/2016|H4N2|//2016
A/ruddy_turnstone/New_Jersey/UGAI17_3019/2017|H9N2|05/27/2017
A/mallard/Ohio/15OS3609/2015|Mixed|08/21/2015
A/mallard/Ohio/15OS4242/2015|H3N8|08/14/2015
A/mallard/Ohio/15OS4266/2015|H3N6|08/21/2015
A/mallard/Ohio/17OS1964/2017|H3N2|08/11/2017
A/mallard/Ohio/15OS3614/2015|H4N8|08/21/2015
A/mallard/Ohio/15OS3609/2015|mixed|08/21/2015
A/mallard/Ohio/15OS3603/2015|H3N8|08/21/2015
A/mallard/Pennsylvania/UGAI15_4737/2015|H3N1|09/08/2015
A/Blue_winged_Teal/Ohio/16OS1039/2016|H2N1|09/04/2016
A/green_winged_teal/Ohio/14OS1964/2014|H3N2|09/06/2014
A/mallard/Ohio/14OS0992/2014|mixed|09/04/2014
A/mallard/Ohio/14OS0573/2014|H4N8|08/22/2014
A/mallard/Ohio/14OS0568/2014|H4N8|08/22/2014
A/mallard/Ohio/14OS0571/2014|mixed|08/22/2014
A/mallard/Ohio/14OS0570/2014|mixed|08/22/2014
A/mallard/Ohio/14OS0435/2014|H4N8|08/26/2014
A/mallard/Ohio/14OS0440/2014|H4N8|08/26/2014
A/mallard/Ohio/14OS0572/2014|H4N8|08/22/2014
A/mallard/Ohio/14OS0438/2014|mixed|08/26/2014
A/mallard/Ohio/14OS0551/2014|H4N8|08/22/2014
A/mallard/Ohio/14OS0441/2014|H4N8|08/26/2014
A/American_black_duck/Ohio/14OS0557/2014|H4N8|08/22/2014
A/mallard/Ohio/14OS0949/2014|mixed|08/26/2014
A/blue_winged_teal/Missouri/15OS4769/2015|H3N2|09/12/2015
A/mallard/Ohio/15OS4278/2015|H3N2|08/21/2015
A/mallard/Ohio/15OS3986/2015|H3N2|08/07/2015
A/mallard_duck/Wisconsin/15OS6906/2015|H1N1|11/14/2015
A/mallard_duck/Wisconsin/15OS7136/2015|H11N9|11/14/2015
A/blue_winged_teal/Iowa/13OS2346/2013|H3N8|09/21/2013
A/blue_winged_teal/Iowa/13OS2345/2013|H3N8|09/21/2013
A/blue_winged_teal/Iowa/13OS2368/2013|H4N8|09/21/2013
A/mallard/Maryland/14OS1307/2014|mixed|08/12/2014
A/mallard/Maryland/14OS1312/2014|H4N6|08/12/2014
A/mallard/Maryland/14OS1309/2014|H4N6|08/12/2014
A/mallard/Maryland/14OS1310/2014|H4N6|08/12/2014
A/mallard/Maryland/14OS1317/2014|H4N6|08/12/2014
A/mallard/Maryland/14OS1321/2014|H4N6|08/12/2014
A/mallard/Maryland/14OS1322/2014|H4N6|08/12/2014
A/environment/Maryland/14OS1379/2014|H4N6|08/26/2014
A/environment/Maryland/14OS1374/2014|H4N6|08/26/2014
A/environment/Maryland/14OS1384/2014|H4N2|08/26/2014
A/environment/Maryland/14OS1364/2014|H4N6|08/26/2014
A/environment/Maryland/14OS1372/2014|mixed|08/26/2014
A/environment/Maryland/14OS1382/2014|H4N6|08/26/2014
A/mallard/Maryland/14OS1329/2014|H10N3|08/12/2014
A/mallard/Maryland/14OS1326/2014|mixed|08/12/2014
A/mallard/Maryland/14OS1302/2014|H10N3|08/12/2014
A/mallard/Maryland/14OS1311/2014|H10N3|08/12/2014
A/mallard/Maryland/14OS1316/2014|H10N3|08/12/2014
A/mallard/Maryland/14OS1306/2014|H10N3|08/12/2014
A/mallard/Maryland/14OS1315/2014|H10N3|08/12/2014
A/mallard/Maryland/14OS1327/2014|H10N3|08/12/2014
A/mallard/Maryland/14OS1328/2014|H10N3|08/12/2014
A/mallard/Maryland/14OS1319/2014|H10N3|08/12/2014
A/mallard/Ohio/14OS3373/2014|H5N2|12/02/2014
A/mallard/Ohio/14OS3379/2014|H5N2|12/07/2014
A/mallard/Ohio/14OS3372/2014|H5N2|12/02/2014
A/green_winged_teal/Ohio/14OS3380/2014|H5N2|12/12/2014
A/mallard/Ohio/14OS3394/2014|H5N2|12/13/2014
A/environment/Maryland/14OS1376/2014|H4N2|08/26/2014
A/environment/Maryland/14OS1381/2014|H4N2|08/26/2014
A/environment/Maryland/14OS1387/2014|mixed|08/26/2014
A/mallard/Ohio/12OS4697/2012|H11N9|10/13/2012
A/mallard/Ohio/17OS1727/2017|H10N8|08/01/2017
A/mallard/Ohio/17OS1892/2017|H10N7|07/26/2017
A/mallard_duck/Ohio/16OS4989/2016|H4N8|11/12/2016
A/green_winged_teal/Ohio/16OS1016/2016|H3N9|09/03/2016
A/mallard_duck/Ohio/16OS0803/2016|H10N7|07/28/2016
A/mallard/New_York/AH0081558/2016|H7N7|01/29/2016
A/northern_pintail/Ohio/15OS5731/2015|H4N6|10/22/2015
A/northern_pintail_duck/Ohio/15OS5737/2015|H4N6|10/23/2015
A/mallard/Ohio/15OS3992/2015|H6N2|08/07/2015
A/mallard/Ohio/15OS4225/2015|H6N2|08/14/2015
A/mallard/Ohio/15OS3999/2015|H6N2|08/11/2015
A/mallard/Ohio/15OS4208/2015|H6N2|08/14/2015
A/mallard/New_York/AH0049315/2016|H7N3|01/25/2016
A/mallard/Illinois/14OS2423/2014|Mixed|10/19/2014
A/mallard/Illinois/14OS2423/2014|mixed|10/19/2014
A/blue_winged_teal/Louisiana/UGAI14_2395/2014|H4N8|09/13/2014
A/mallard/Wisconsin/14OS2282/2014|H4N8|10/05/2014
A/mallard/Ohio/14OS3201/2014|H4N6|11/18/2014
A/blue_winged_teal/Texas/AI11_3655/2011|mixed|09/17/2011
A/blue_winged_teal/Texas/AI11_3233/2011|mixed|09/17/2011
A/blue_winged_teal/Texas/AI11_3100/2011|H3N6|09/17/2011
A/gadwall/Mississippi/11OS6084/2011|H11N2|12/10/2011
A/northern_shoveler/Mississippi/11OS6077/2011|H11N2|12/10/2011
A/mallard/Wisconsin/11OS3157/2011|H3N2|09/24/2011
A/mallard/Wisconsin/11OS3158/2011|H3N2|09/24/2011
A/mallard/Wisconsin/11OS4238/2011|H3N2|10/23/2011
A/mallard/Wisconsin/11OS3467/2011|mixed|10/16/2011
A/mallard/Wisconsin/11OS3079/2011|H3N2|09/24/2011
A/mallard/Minnesota/AI09_1829/2009|H3N8|07/28/2009
A/mallard/Minnesota/AI09_3047/2009|mixed|09/07/2009
A/mallard/Minnesota/AI08_5469/2008|H7N3|09/25/2008
A/mallard/Minnesota/Sg_00868/2008|H3N8|09/03/2008
A/mallard/Minnesota/Sg_00928/2008|mixed|09/16/2008
A/mallard/Minnesota/Sg_00616/2008|H4N8|07/29/2008
2008
2010
2012
2014
2016
2018
2020

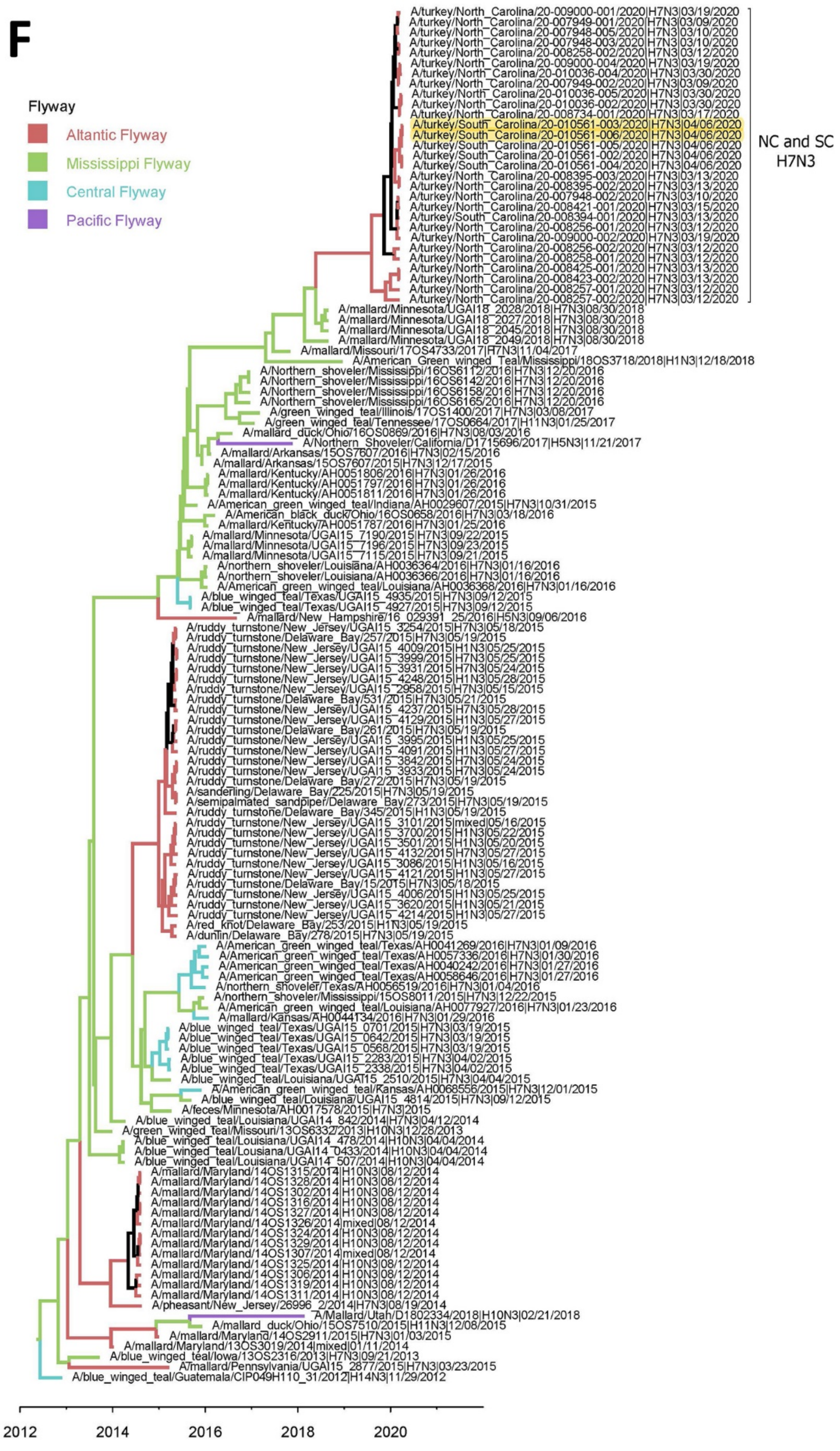
F
Flyway
Altantic Flyway
Mississippi Flyway
Central Flyway
Pacific Flyway
NC and SC H7N3
A/turkey/North_Carolina/20-009000-001/2020|H7N3|03/19/2020
A/turkey/North_Carolina/20-007949-001/2020|H7N3|03/09/2020
A/turkey/North_Carolina/20-007948-005/2020|H7N3|03/10/2020
A/turkey/North_Carolina/20-007948-003/2020|H7N3|03/10/2020
A/turkey/North_Carolina/20-008258-002/2020|H7N3|03/12/2020
A/turkey/North_Carolina/20-009000-004/2020|H7N3|03/19/2020
A/turkey/North_Carolina/20-010036-004/2020|H7N3|03/30/2020
A/turkey/North_Carolina/20-007949-002/2020|H7N3|03/09/2020
A/turkey/North_Carolina/20-010036-005/2020|H7N3|03/30/2020
A/turkey/North_Carolina/20-010036-002/2020|H7N3|03/30/2020
A/turkey/North_Carolina/20-008734-001/2020|H7N3|03/17/2020
A/turkey/South_Carolina/20-010561-003/2020|H7N3|04/06/2020
A/turkey/South_Carolina/20-010561-006/2020|H7N3|04/06/2020
A/turkey/South_Carolina/20-010561-005/2020|H7N3|04/06/2020
A/turkey/South_Carolina/20-010561-002/2020|H7N3|04/06/2020
A/turkey/South_Carolina/20-010561-004/2020|H7N3|04/06/2020
A/turkey/North_Carolina/20-008395-003/2020|H7N3|03/13/2020
A/turkey/North_Carolina/20-008395-002/2020|H7N3|03/13/2020
A/turkey/North_Carolina/20-007948-002/2020|H7N3|03/10/2020
A/turkey/North_Carolina/20-008421-001/2020|H7N3|03/15/2020
A/turkey/South_Carolina/20-008394-001/2020|H7N3|03/13/2020
A/turkey/North_Carolina/20-008256-001/2020|H7N3|03/12/2020
A/turkey/North_Carolina/20-009000-002/2020|H7N3|03/19/2020
A/turkey/North_Carolina/20-008256-002/2020|H7N3|03/12/2020
A/turkey/North_Carolina/20-008258-001/2020|H7N3|03/12/2020
A/turkey/North_Carolina/20-008425-001/2020|H7N3|03/13/2020
A/turkey/North_Carolina/20-008423-002/2020|H7N3|03/13/2020
A/turkey/North_Carolina/20-008257-001/2020|H7N3|03/12/2020
A/turkey/North_Carolina/20-008257-002/2020|H7N3|03/12/2020
A/mallard/Minnesota/UGAI18_2028/2018|H7N3|08/30/2018
A/mallard/Minnesota/UGAI18_2027/2018|H7N3|08/30/2018
A/mallard/Minnesota/UGAI18_2045/2018|H7N3|08/30/2018
A/mallard/Minnesota/UGAI18_2049/2018|H7N3|08/30/2018
A/mallard/Missouri/17OS4733/2017|H7N3|11/04/2017
A/American_Green_winged_Teal/Mississippi/18OS3718/2018|H1N3|12/18/2018
A/Northern_shoveler/Mississippi/16OS6112/2016|H7N3|12/20/2016
A/Northern_shoveler/Mississippi/16OS6142/2016|H7N3|12/20/2016
A/Northern_shoveler/Mississippi/16OS6158/2016|H7N3|12/20/2016
A/Northern_shoveler/Mississippi/16OS6165/2016|H7N3|12/20/2016
A/green_winged_teal/Illinois/17OS1400/2017|H7N3|03/08/2017
A/green_winged_teal/Tennessee/17OS0664/2017|H11N3|01/25/2017
A/mallard_duck/Ohio/16OS0869/2016|H7N3|08/03/2016
A/Northern_Shoveler/California/D1715696/2017|H5N3|11/21/2017
A/mallard/Arkansas/15OS7607/2016|H7N3|02/15/2016
A/mallard/Arkansas/15OS7607/2015|H7N3|12/17/2015
A/mallard/Kentucky/AH0051806/2016|H7N3|01/26/2016
A/mallard/Kentucky/AH0051797/2016|H7N3|01/26/2016
A/mallard/Kentucky/AH0051811/2016|H7N3|01/26/2016
A/American_green_winged_teal/Indiana/AH0029607/2015|H7N3|10/31/2015
A/American_black_duck/Ohio/16OS0658/2016|H7N3|03/18/2016
A/mallard/Kentucky/AH0051787/2016|H7N3|01/25/2016
A/mallard/Minnesota/UGAI15_7190/2015|H7N3|09/22/2015
A/mallard/Minnesota/UGAI15_7196/2015|H7N3|09/23/2015
A/mallard/Minnesota/UGAI15_7115/2015|H7N3|09/21/2015
A/northern_shoveler/Louisiana/AH0036364/2016|H7N3|01/16/2016
A/northern_shoveler/Louisiana/AH0036366/2016|H7N3|01/16/2016
A/American_green_winged_teal/Louisiana/AH0036368/2016|H7N3|01/16/2016
A/blue_winged_teal/Texas/UGAI15_4935/2015|H7N3|09/12/2015
A/blue_winged_teal/Texas/UGAI15_4927/2015|H7N3|09/12/2015
A/mallard/New_Hampshire/16_029391_25/2016|H5N3|09/06/2016
A/ruddy_turnstone/New_Jersey/UGAI15_3254/2015|H7N3|05/18/2015
A/ruddy_turnstone/Delaware_Bay/257/2015|H7N3|05/19/2015
A/ruddy_turnstone/New_Jersey/UGAI15_4009/2015|H1N3|05/25/2015
A/ruddy_turnstone/New_Jersey/UGAI15_3999/2015|H1N3|05/25/2015
A/ruddy_turnstone/New_Jersey/UGAI15_3931/2015|H7N3|05/24/2015
A/ruddy_turnstone/New_Jersey/UGAI15_4248/2015|H1N3|05/28/2015
A/ruddy_turnstone/New_Jersey/UGAI15_2958/2015|H7N3|05/15/2015
A/ruddy_turnstone/Delaware_Bay/531/2015|H7N3|05/21/2015
A/ruddy_turnstone/New_Jersey/UGAI15_4237/2015|H7N3|05/28/2015
A/ruddy_turnstone/New_Jersey/UGAI15_4129/2015|H1N3|05/27/2015
A/ruddy_turnstone/Delaware_Bay/261/2015|H7N3|05/19/2015
A/ruddy_turnstone/New_Jersey/UGAI15_3995/2015|H1N3|05/25/2015
A/ruddy_turnstone/New_Jersey/UGAI15_4091/2015|H1N3|05/27/2015
A/ruddy_turnstone/New_Jersey/UGAI15_3842/2015|H7N3|05/24/2015
A/ruddy_turnstone/New_Jersey/UGAI15_3933/2015|H7N3|05/24/2015
A/ruddy_turnstone/Delaware_Bay/272/2015|H7N3|05/19/2015
A/sanderling/Delaware_Bay/225/2015|H7N3|05/19/2015
A/semipalmated_sandpiper/Delaware_Bay/273/2015|H7N3|05/19/2015
A/ruddy_turnstone/Delaware_Bay/345/2015|H1N3|05/19/2015
A/ruddy_turnstone/New_Jersey/UGAI15_3101/2015|mixed|05/16/2015
A/ruddy_turnstone/New_Jersey/UGAI15_3700/2015|H1N3|05/22/2015
A/ruddy_turnstone/New_Jersey/UGAI15_3501/2015|H1N3|05/20/2015
A/ruddy_turnstone/New_Jersey/UGAI15_4132/2015|H7N3|05/27/2015
A/ruddy_turnstone/New_Jersey/UGAI15_3086/2015|H1N3|05/16/2015
A/ruddy_turnstone/New_Jersey/UGAI15_4121/2015|H1N3|05/27/2015
A/ruddy_turnstone/Delaware_Bay/15/2015|H7N3|05/18/2015
A/ruddy_turnstone/New_Jersey/UGAI15_4006/2015|H1N3|05/25/2015
A/ruddy_turnstone/New_Jersey/UGAI15_3620/2015|H1N3|05/21/2015
A/ruddy_turnstone/New_Jersey/UGAI15_4214/2015|H1N3|05/27/2015
A/red_knot/Delaware_Bay/253/2015|H1N3|05/19/2015
A/dunlin/Delaware_Bay/278/2015|H7N3|05/19/2015
A/American_green_winged_teal/Texas/AH0041269/2016|H7N3|01/09/2016
A/American_green_winged_teal/Texas/AH0057336/2016|H7N3|01/30/2016
A/American_green_winged_teal/Texas/AH0040242/2016|H7N3|01/27/2016
A/American_green_winged_teal/Texas/AH0058646/2016|H7N3|01/27/2016
A/northern_shoveler/Texas/AH0056519/2016|H7N3|01/04/2016
A/northern_shoveler/Mississippi/15OS8011/2015|H7N3|12/22/2015
A/American_green_winged_teal/Louisiana/AH0077927/2016|H7N3|01/23/2016
A/mallard/Kansas/AH0044134/2016|H7N3|01/29/2016
A/blue_winged_teal/Texas/UGAI15_0701/2015|H7N3|03/19/2015
A/blue_winged_teal/Texas/UGAI15_0642/2015|H7N3|03/19/2015
A/blue_winged_teal/Texas/UGAI15_0568/2015|H7N3|03/19/2015
A/blue_winged_teal/Texas/UGAI15_2283/2015|H7N3|04/02/2015
A/blue_winged_teal/Texas/UGAI15_2338/2015|H7N3|04/02/2015
A/blue_winged_teal/Louisiana/UGAI15_2510/2015|H7N3|04/04/2015
A/American_green_winged_teal/Kansas/AH0068556/2015|H7N3|12/01/2015
A/blue_winged_teal/Louisiana/UGAI15_4814/2015|H7N3|09/12/2015
A/feces/Minnesota/AH0017578/2015|H7N3|2015
A/blue_winged_teal/Louisiana/UGAI14_842/2014|H7N3|04/12/2014
A/green_winged_teal/Missouri/13OS6332/2013|H10N3|12/28/2013
A/blue_winged_teal/Louisiana/UGAI14_478/2014|H10N3|04/04/2014
A/blue_winged_teal/Lousiana/UGAI14_0433/2014|H10N3|04/04/2014
A/blue_winged_teal/Lousiana/UGAI14_507/2014|H10N3|04/04/2014
A/mallard/Maryland/14OS1315/2014|H10N3|08/12/2014
A/mallard/Maryland/14OS1328/2014|H10N3|08/12/2014
A/mallard/Maryland/14OS1302/2014|H10N3|08/12/2014
A/mallard/Maryland/14OS1316/2014|H10N3|08/12/2014
A/mallard/Maryland/14OS1327/2014|H10N3|08/12/2014
A/mallard/Maryland/14OS1326/2014|mixed|08/12/2014
A/mallard/Maryland/14OS1324/2014|H10N3|08/12/2014
A/mallard/Maryland/14OS1329/2014|H10N3|08/12/2014
A/mallard/Maryland/14OS1307/2014|mixed|08/12/2014
A/mallard/Maryland/14OS1325/2014|H10N3|08/12/2014
A/mallard/Maryland/14OS1306/2014|H10N3|08/12/2014
A/mallard/Maryland/14OS1319/2014|H10N3|08/12/2014
A/mallard/Maryland/14OS1311/2014|H10N3|08/12/2014
A/pheasant/New_Jersey/26996_2/2014|H7N3|08/19/2014
A/Mallard/Utah/D1802334/2018|H10N3|02/21/2018
A/mallard_duck/Ohio/15OS7510/2015|H11N3|12/08/2015
A/mallard/Maryland/14OS2911/2015|H7N3|01/03/2015
A/mallard/Maryland/13OS3019/2014|mixed|01/11/2014
A/blue_winged_teal/Iowa/13OS2316/2013|H7N3|09/21/2013
A/mallard/Pennsylvania/UGAI15_2877/2015|H7N3|03/23/2015
A/blue_winged_teal/Guatemala/CIP049H110_31/2012|H14N3|11/29/2012
2012
2014
2016
2018
2020

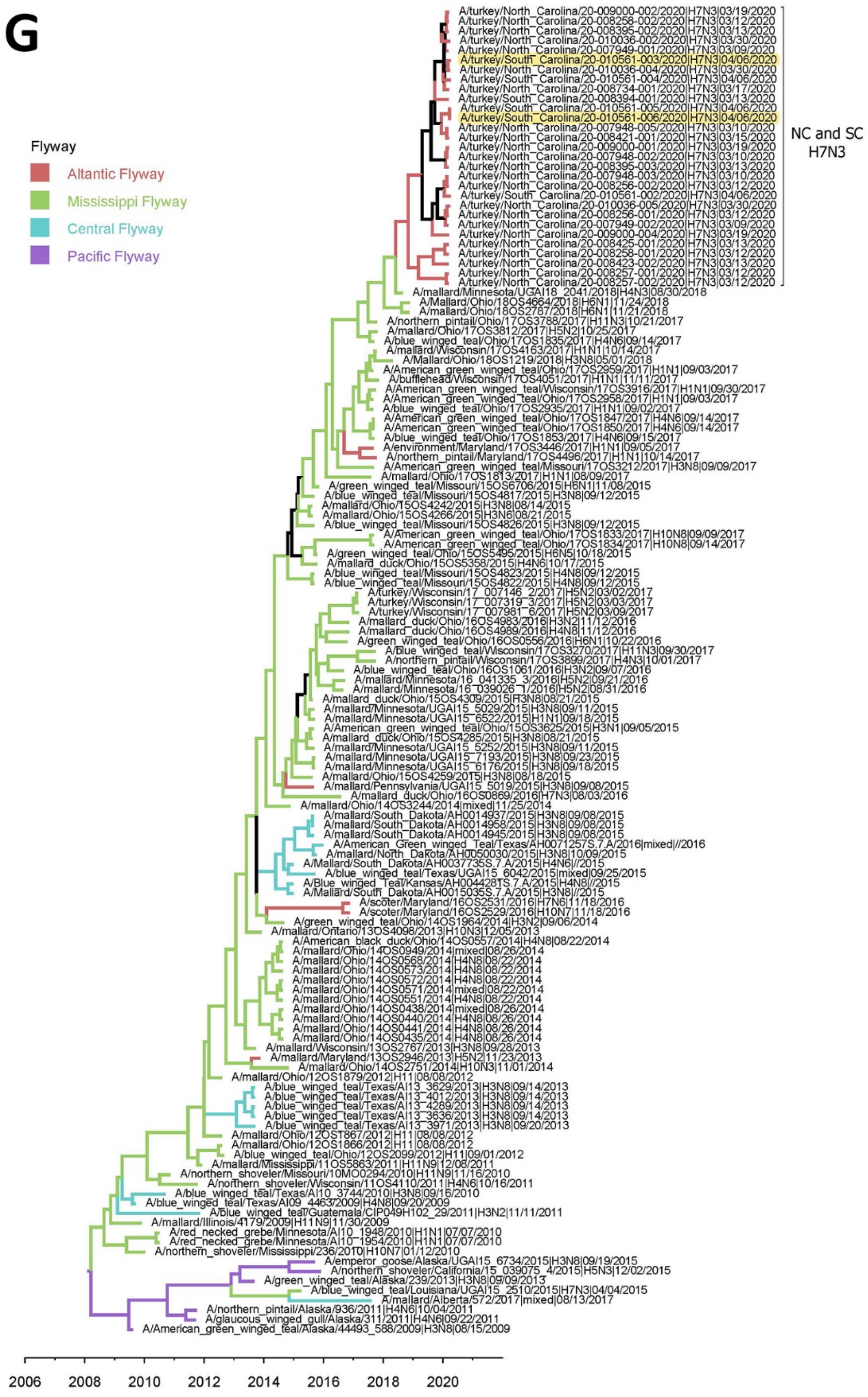
G
Flyway
Altantic Flyway
Mississippi Flyway
Central Flyway
Pacific Flyway
NC and SC
H7N3
A/turkey/North_Carolina/20-009000-002/2020|H7N3|03/19/2020
A/turkey/North_Carolina/20-008258-002/2020|H7N3|03/12/2020
A/turkey/North_Carolina/20-008395-002/2020|H7N3|03/13/2020
A/turkey/North_Carolina/20-010036-002/2020|H7N3|03/30/2020
A/turkey/North_Carolina/20-007949-001/2020|H7N3|03/09/2020
A/turkey/South_Carolina/20-010561-003/2020|H7N3|04/06/2020
A/turkey/North_Carolina/20-010036-004/2020|H7N3|03/30/2020
A/turkey/South_Carolina/20-010561-004/2020|H7N3|04/06/2020
A/turkey/North_Carolina/20-008734-001/2020|H7N3|03/17/2020
A/turkey/South_Carolina/20-008394-001/2020|H7N3|03/13/2020
A/turkey/South_Carolina/20-010561-005/2020|H7N3|04/06/2020
A/turkey/South_Carolina/20-010561-006/2020|H7N3|04/06/2020
A/turkey/North_Carolina/20-007948-005/2020|H7N3|03/10/2020
A/turkey/North_Carolina/20-008421-001/2020|H7N3|03/15/2020
A/turkey/North_Carolina/20-009000-001/2020|H7N3|03/19/2020
A/turkey/North_Carolina/20-007948-002/2020|H7N3|03/10/2020
A/turkey/North_Carolina/20-008395-003/2020|H7N3|03/13/2020
A/turkey/North_Carolina/20-007948-003/2020|H7N3|03/10/2020
A/turkey/North_Carolina/20-008256-002/2020|H7N3|03/12/2020
A/turkey/South_Carolina/20-010561-002/2020|H7N3|04/06/2020
A/turkey/North_Carolina/20-010036-005/2020|H7N3|03/30/2020
A/turkey/North_Carolina/20-008256-001/2020|H7N3|03/12/2020
A/turkey/North_Carolina/20-007949-002/2020|H7N3|03/09/2020
A/turkey/North_Carolina/20-009000-004/2020|H7N3|03/19/2020
A/turkey/North_Carolina/20-008425-001/2020|H7N3|03/13/2020
A/turkey/North_Carolina/20-008258-001/2020|H7N3|03/12/2020
A/turkey/North_Carolina/20-008423-002/2020|H7N3|03/13/2020
A/turkey/North_Carolina/20-008257-001/2020|H7N3|03/12/2020
A/turkey/North_Carolina/20-008257-002/2020|H7N3|03/12/2020
A/mallard/Minnesota/UGAI18_2041/2018|H4N3|08/30/2018
A/Mallard/Ohio/18OS4664/2018|H6N1|11/24/2018
A/mallard/Ohio/18OS2787/2018|H6N1|11/21/2018
A/northern_pintail/Ohio/17OS3788/2017|H11N3|10/21/2017
A/mallard/Ohio/17OS3812/2017|H5N2|10/25/2017
A/blue_winged_teal/Ohio/17OS1835/2017|H4N6|09/14/2017
A/mallard/Wisconsin/17OS4163/2017|H1N1|10/14/2017
A/Mallard/Ohio/18OS1219/2018|H3N8|05/01/2018
A/American_green_winged_teal/Ohio/17OS2959/2017|H1N1|09/03/2017
A/bufflehead/Wisconsin/17OS4051/2017|H1N1|11/11/2017
A/American_green_winged_teal/Wisconsin/17OS3916/2017|H1N1|09/30/2017
A/American_green_winged_teal/Ohio/17OS2958/2017|H1N1|09/03/2017
A/blue_winged_teal/Ohio/17OS2935/2017|H1N1|09/02/2017
A/American_green_winged_teal/Ohio/17OS1847/2017|H4N6|09/14/2017
A/American_green_winged_teal/Ohio/17OS1850/2017|H4N6|09/14/2017
A/blue_winged_teal/Ohio/17OS1853/2017|H4N6|09/15/2017
A/environment/Maryland/17OS3446/2017|H1N1|09/05/2017
A/northern_pintail/Maryland/17OS4496/2017|H1N1|10/14/2017
A/American_green_winged_teal/Missouri/17OS3212/2017|H3N8|09/09/2017
A/mallard/Ohio/17OS1813/2017|H1N1|08/09/2017
A/green_winged_teal/Missouri/15OS6706/2015|H6N1|11/08/2015
A/blue_winged_teal/Missouri/15OS4817/2015|H3N8|09/12/2015
A/mallard/Ohio/15OS4242/2015|H3N8|08/14/2015
A/mallard/Ohio/15OS4266/2015|H3N6|08/21/2015
A/blue_winged_teal/Missouri/15OS4826/2015|H3N8|09/12/2015
A/American_green_winged_teal/Ohio/17OS1833/2017|H10N8|09/09/2017
A/American_green_winged_teal/Ohio/17OS1834/2017|H10N8|09/14/2017
A/green_winged_teal/Ohio/15OS5495/2015|H6N5|10/18/2015
A/mallard_duck/Ohio/15OS5358/2015|H4N6|10/17/2015
A/blue_winged_teal/Missouri/15OS4823/2015|H4N8|09/12/2015
A/blue_winged_teal/Missouri/15OS4822/2015|H4N8|09/12/2015
A/turkey/Wisconsin/17_007146_2/2017|H5N2|03/02/2017
A/turkey/Wisconsin/17_007319_3/2017|H5N2|03/03/2017
A/turkey/Wisconsin/17_007981_6/2017|H5N2|03/09/2017
A/mallard_duck/Ohio/16OS4983/2016|H3N2|11/12/2016
A/mallard_duck/Ohio/16OS4989/2016|H4N8|11/12/2016
A/green_winged_teal/Ohio/16OS0556/2016|H6N1|10/22/2016
A/blue_winged_teal/Wisconsin/17OS3270/2017|H11N3|09/30/2017
A/northern_pintail/Wisconsin/17OS3899/2017|H4N3|10/01/2017
A/blue_winged_teal/Ohio/16OS1061/2016|H3N2|09/07/2016
A/mallard/Minnesota/16_041335_3/2016|H5N2|09/21/2016
A/mallard/Minnesota/16_039026_1/2016|H5N2|08/31/2016
A/mallard_duck/Ohio/15OS4309/2015|H3N8|08/21/2015
A/mallard/Minnesota/UGAI15_5029/2015|H3N8|09/11/2015
A/mallard/Minnesota/UGAI15_6522/2015|H1N1|09/18/2015
A/American_green_winged_teal/Ohio/15OS3625/2015|H3N1|09/05/2015
A/mallard_duck/Ohio/15OS4285/2015|H3N8|08/21/2015
A/mallard/Minnesota/UGAI15_5252/2015|H3N8|09/11/2015
A/mallard/Minnesota/UGAI15_7193/2015|H3N8|09/23/2015
A/mallard/Minnesota/UGAI15_6176/2015|H3N8|09/18/2015
A/mallard/Ohio/15OS4259/2015|H3N8|08/18/2015
A/mallard/Pennsylvania/UGAI15_5019/2015|H3N8|09/08/2015
A/mallard_duck/Ohio/16OS0869/2016|H7N3|08/03/2016
A/mallard/Ohio/14OS3244/2014|mixed|11/25/2014
A/mallard/South_Dakota/AH0014937/2015|H3N8|09/08/2015
A/mallard/South_Dakota/AH0014958/2015|H3N8|09/08/2015
A/mallard/South_Dakota/AH0014945/2015|H3N8|09/08/2015
A/American_Green_winged_Teal/Texas/AH0071257S.7.A/2016|mixed|//2016
A/mallard/North_Dakota/AH0050030/2015|H3N8|10/09/2015
A/Mallard/South_Dakota/AH0037735S.7.A/2015|H4N6|//2015
A/blue_winged_teal/Texas/UGAI15_6042/2015|mixed|09/25/2015
A/Blue_winged_Teal/Kansas/AH004428TS.7.A/2015|H4N8|//2015
A/Mallard/South_Dakota/AH0015035S.7.A/2015|H3N8|//2015
A/scoter/Maryland/16OS2531/2016|H7N6|11/18/2016
A/scoter/Maryland/16OS2529/2016|H10N7|11/18/2016
A/green_winged_teal/Ohio/14OS1964/2014|H3N2|09/06/2014
A/mallard/Ontario/13OS4098/2013|H10N3|12/05/2013
A/American_black_duck/Ohio/14OS0557/2014|H4N8|08/22/2014
A/mallard/Ohio/14OS0949/2014|mixed|08/26/2014
A/mallard/Ohio/14OS0568/2014|H4N8|08/22/2014
A/mallard/Ohio/14OS0573/2014|H4N8|08/22/2014
A/mallard/Ohio/14OS0572/2014|H4N8|08/22/2014
A/mallard/Ohio/14OS0571/2014|mixed|08/22/2014
A/mallard/Ohio/14OS0551/2014|H4N8|08/22/2014
A/mallard/Ohio/14OS0438/2014|mixed|08/26/2014
A/mallard/Ohio/14OS0440/2014|H4N8|08/26/2014
A/mallard/Ohio/14OS0441/2014|H4N8|08/26/2014
A/mallard/Ohio/14OS0435/2014|H4N8|08/26/2014
A/mallard/Wisconsin/13OS2767/2013|H3N8|09/28/2013
A/mallard/Maryland/13OS2946/2013|H5N2|11/23/2013
A/mallard/Ohio/14OS2751/2014|H10N3|11/01/2014
A/mallard/Ohio/12OS1879/2012|H11|08/08/2012
A/blue_winged_teal/Texas/AI13_3629/2013|H3N8|09/14/2013
A/blue_winged_teal/Texas/AI13_4012/2013|H3N8|09/14/2013
A/blue_winged_teal/Texas/AI13_4289/2013|H3N8|09/14/2013
A/blue_winged_teal/Texas/AI13_3636/2013|H3N8|09/14/2013
A/blue_winged_teal/Texas/AI13_3971/2013|H3N8|09/20/2013
A/mallard/Ohio/12OST867/2012|H11|08/08/2012
A/mallard/Ohio/12OS1866/2012|H11|08/08/2012
A/blue_winged_teal/Ohio/12OS2099/2012|H11|09/01/2012
A/mallard/Mississippi/11OS5863/2011|H11N9|12/08/2011
A/northern_shoveler/Missouri/10MO0294/2010|H11N9|11/16/2010
A/northern_shoveler/Wisconsin/11OS4110/2011|H4N6|10/16/2011
A/blue_winged_teal/Texas/AI10_3744/2010|H3N8|09/16/2010
A/blue_winged_teal/Texas/AI09_4463/2009|H4N8|09/20/2009
A/blue_winged_teal/Guatemala/CIP049H102_29/2011|H3N2|11/11/2011
A/mallard/Illinois/4179/2009|H11N9|11/30/2009
A/red_necked_grebe/Minnesota/AI10_1948/2010|H1N1|07/07/2010
A/red_necked_grebe/Minnesota/AI10_1954/2010|H1N1|07/07/2010
A/northern_shoveler/Mississippi/236/2010|H10N7|01/12/2010
A/emperor_goose/Alaska/UGAI15_6734/2015|H3N8|09/19/2015
A/northern_shoveler/California/15_039075_4/2015|H5N3|12/02/2015
A/green_winged_teal/Alaska/239/2013|H3N8|09/09/2013
A/blue_winged_teal/Louisiana/UGAI15_2510/2015|H7N3|04/04/2015
A/mallard/Alberta/572/2017|mixed|08/13/2017
A/northern_pintail/Alaska/936/2011|H4N6|10/04/2011
A/glaucous_winged_gull/Alaska/311/2011|H4N6|09/22/2011
A/American_green_winged_teal/Alaska/44493_588/2009|H3N8|08/15/2009
2006
2008
2010
2012
2014
2016
2018
2020

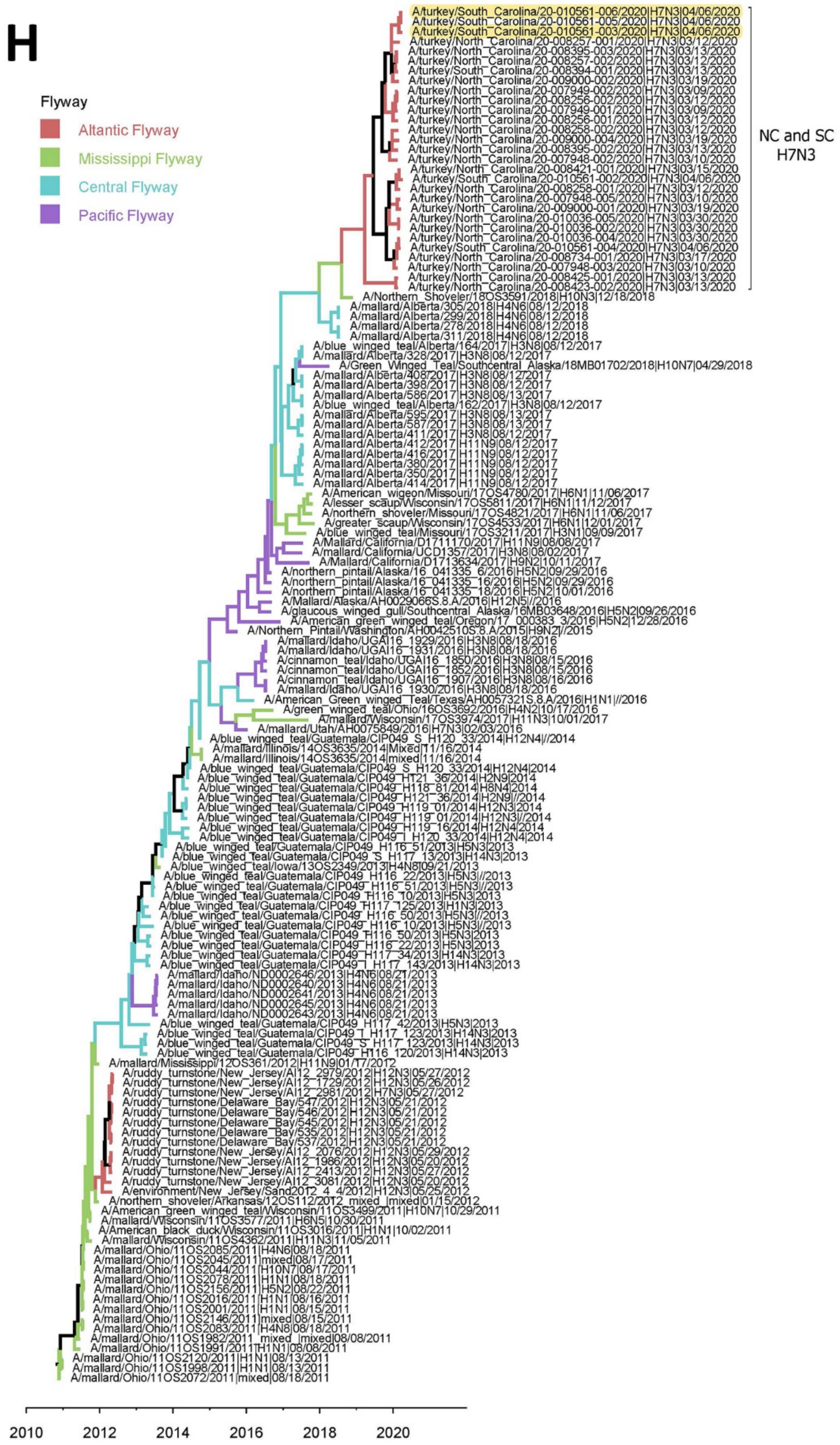

Appendix Figure 2. Time-scaled Bayesian phylogenetic trees. Branches are colored according to discrete geographic areas including 4 administrative bird flyways in North America (Atlantic, Mississippi,

Central, and Pacific) according to the flyway boundaries established by the US Fish and Wildlife Service. Blue node bars represent 95% Bayesian credible intervals (BCI). A) polymerase basic 2 (PB2); B) PB1; C) polymerase (PA); D) hemagglutinin (HA); E) nucleoprotein (NP); F) neuraminidase (NA); G) matrix (M); H) nonstructural (NS).
